# Distributed Intelligent Battery Management System Using a Real-World Cloud Computing System

**DOI:** 10.3390/s23073417

**Published:** 2023-03-24

**Authors:** Emilio García, Eduardo Quiles, Antonio Correcher

**Affiliations:** Instituto de Automática e Informática Industrial, Universitat Politècnica de València, 46022 Valencia, Spain

**Keywords:** battery management system, SOC, SOH, temperature sensor, charge regulator, cloud computing, ESS

## Abstract

In this work, a decentralized but synchronized real-world system for smart battery management was designed by using a general controller with cloud computing capability, four charge regulators, and a set of sensorized battery monitors with networking and Bluetooth capabilities. Currently, for real-world applications, battery management systems (BMSs) can be used in the form of distributed control systems where general controllers, charge regulators, and smart monitors and sensors are integrated, such as those proposed in this work, which allow more precise estimations of a large set of important parameters, such as the state of charge (SOC), state of health (SOH), current, voltage, and temperature, seeking the safety and the extension of the useful life of energy storage systems based on battery banks. The system used is a paradigmatic real-world example of the so-called intelligent battery management systems. One of the contributions made in this work is the realization of a distributed design of a BMS, which adds the benefit of increased system security compared to a fully centralized BMS structure. Another research contribution made in this work is the development of a methodical modeling procedure based on Petri Nets, which establishes, in a visible, organized, and precise way, the set of conditions that will determine the operation of the BMS. If this modeling is not carried out, the threshold values and their conditions remain scattered, not very transparent, and difficult to deal with in an aggregate way.

## 1. Introduction

At present, a very worrying energy crisis is affecting socio-political, economic, and energy supply stability on a global scale. For this last aspect, it is particularly necessary to intensify the promotion of an energy self-sufficiency policy based on renewable energies.

One of the objectives for further increasing the competitiveness of renewable energies must be based on the improvement of energy storage systems (ESSs). In particular, it is important to improve the operating and maintenance costs of the installations based on the application of advanced methods for the monitoring and predictive diagnosis of failures, aimed at extending the useful life of the batteries. The price of a photovoltaic (PV) system is significantly impacted by the reduced battery life. More than 40% of the costs over the course of a PV system’s life cycle are attributable to the battery [1]. System reliability will improve, and operating costs will be significantly reduced as the battery’s lifespan increases [2,3]. By avoiding hazardous operating conditions such as deep discharge and overcharging, batteries’ lifespans can be increased.

A battery can offer many years of dependable service if it is properly designed, constructed, and maintained. A brand-new battery may not initially operate at full capacity. During the first few years of use, the capacity usually increases, peaks, and then drops until the battery reaches the end of its useful life. A lead–acid battery is generally considered to have reached the end of its useful life when its capacity reaches 80% of its rated capacity. Below 80%, the rate of battery deterioration quickens, making it more susceptible to a mechanical shock or a rapid failure induced by a high discharge rate. It should be noted that a battery will eventually lose its capacity even in perfect circumstances [4].

The storage capacity and useful life of batteries are significantly influenced by the appropriate management of their use, which is necessary to achieve the energy storage system’s maximum availability [5]. Batteries can be used differently by condition-monitoring devices to increase their lifespan. Additionally, they can regulate the state of the batteries, allowing the application of predictive maintenance approaches to decide when to replace them [6,7]. They can also estimate the quantity of energy stored in the batteries to plan power usage and charging cycles.

### 1.1. Maintenance Particularities of Each Type of Battery

The useful life of batteries depends on many factors, and it is known that it can be shortened by both overcharging and undercharging and overloading. It can also be highly detrimental if the current discharges are too deep under conditions of high ambient temperatures or due to the confined space of the installation site. This phenomenon is general and affects different types of batteries, either Li-ion type [8] or Valve-Regulated Lead–Acid (VRLA)-type batteries (gel or Absorbent Glass Mat (AGM)), which, due to their comparatively low cost, are still widely used [9].

For VRLA (gel or AGM) batteries, overcharging can cause gassing, which dries out the electrolyte and raises the internal resistance, leading to irreparable damage. When the charging voltage of 12 V batteries approaches 15 V, flat-plate VRLA batteries begin to leak water [1].

It should be noted that Li-ion batteries are particularly expensive and that they are also susceptible to irreversible damage from excessive charging or discharging. When the system is not in use, various components (alarm systems, relays, standby current from specific loads, current drain from battery chargers, etc.) gradually drain the battery. Since Li-ion batteries have a much higher charging efficiency than lead–acid batteries, it is advised to set the charging efficiency factor to 99 percent [10].

LiFePO4 batteries can generally use the factory-programmed “charging parameters” as well. When the current falls below a set threshold, some battery chargers stop charging. This limit must be exceeded by the tail current. LiFePO4 batteries outperform lead–acid batteries at high discharge rates. The Peukert exponent should be set to 1.05 [10] unless the battery manufacturer specifies otherwise.

When the system is not in use, the battery should be isolated in case there is any uncertainty regarding potential residual current consumption by opening its circuit breaker, removing the battery fuse(s), or disconnecting the battery’s positive terminal. If the system has been completely discharged and the low-voltage disconnection of the cells has taken place, the residual discharge current is particularly hazardous. A Li-ion battery still has a reserve of about 1 Ah for every 100 Ah of capacity after being disconnected due to the low cell voltage. If the battery’s reserve capacity is reduced, the battery will suffer damage. If the system is left in a discharged state for more than 10 days, a residual current of 4 mA, for instance, can destroy a 100 Ah battery (4 mA × 24 h × 10 days = 0.96 Ah). It is especially advised in these circumstances to use devices with low current consumption [10].

### 1.2. BMS Applications

By managing the battery with an intelligent battery management system (BMS), information is received that will allow major degradation problems to be avoided. The amortization of a BMS is quickly realized by contributing to the prolongation of battery life.

It would be desirable if a BMS could have easy access to the innards of commonly used batteries to protect the drive battery’s individual cells and to increase the service life as well as the cycle number. This possibility would be very interesting for most types of batteries. However, this is not feasible for practically all commercial batteries, except in the case of laboratory tests. In this final instance, the BMS measures the control parameters: the battery current, temperature, and cell voltage. The nominal voltage of a common battery cell is 3.6 V, with a maximum end-of-charge voltage of 4.2 V and a minimum end-of-discharge voltage of 2.5 V. Unrepairable damage, such as capacity loss and an increase in self-discharging, is brought on by high discharging (<2.5 V). Overvoltage (>4.2 V) can cause spontaneous self-ignition, which is dangerous. When temperatures and voltages are excessively high while charging, there is a significant risk of capacity loss. A typical battery has a lifespan of 500 to 1000 cycles when used properly before losing 20% of its initial capacity.

The forecasting of a battery’s state of charge (SOC) and state of health (SOH) is possible in part by monitoring the cell voltage, current, and temperature. SOC refers to the battery’s present level of charge in relation to its maximum capacity. SOH describes the battery’s present health in comparison to a brand-new battery [11].

In summary, among the main functions to be performed on batteries by a BMS are charge and discharge control [12], thermal management [13], battery equalization [14], fault diagnosis [15,16,17], data acquisition, communication [18], and the estimation of the battery’s state of charge [19,20], state of energy (SoE) [21], state of power (SoP) [22], and state of health (SoH) [23]. Especially in top-priority safety-critical cases, they require the application of safeguarding functions to the system, including battery disconnection operations from generation or consumption sources [24].

Recent research [25,26,27] has reported smart BMS systems to implement specific functions in critical applications. The aircraft industry, automotive sector [28], and renewable energy grid integration applications [26] are fields where the BMS plays an essential role in system performance. Aircraft electrification’s modern challenges [29] involve migrating from hydraulic or pneumatic onboard systems to electrical systems. This is the so-called “More electric Aircraft” (MEA) research field. European projects such as I-PRIMES [30], MOET [31], EPOCAl [32], and ENIGMA [27] tackle the control of the electrical system. The optimal operation of the batteries leads to efficient battery sizing, which involves less weight, a critical aspect in airplanes. Therefore, the design of more innovative MEA control systems [29] (including BMSs) is necessary. Electric vehicles are also a hot topic in BMS research [33,34]. As in the aircraft industry, optimal battery sizing reduces the car’s weight so that autonomy can be increased with optimal BMS development.

### 1.3. Battery Modeling

Numerous researchers [35] have investigated the dynamic behavior of battery operation. Lead–acid batteries were used to power the models created years ago [36,37,38], but lithium and nickel–cadmium batteries are similar in some ways. Lead–acid chemistry is similar to nickel–cadmium (NiCd) chemistry in that an electrolyte contains two different metals. Unlike sulfuric acid in lead–acid batteries, potassium hydroxide (KOH) does not enter the reaction in NiCd batteries. Because the positive and negative plates alternate while being submerged in an electrolyte, the manufacturing process is comparable to lead–acid batteries [39]. A summary of the mathematical lithium and NiCd battery models created at the University of South Carolina is provided in [40]. Numerous authors have developed in-depth dynamic models of lithium batteries. These range from straightforward models with resistance (R) or parallel-resistance capacitors (RC) [41,42,43] to more intricate models with phase change components and coils [44,45]. Placing these components in series and incorporating particularities to achieve higher levels of adjustment in the electrical behavior of the battery was the researchers’ primary method of operation [46,47].

Other mathematical models have been developed in addition to these electrical models to estimate the parametric variations depending on the values associated with the time of use/disuse and changes in battery temperature [48]. The remainder of the battery’s useful life (RUL) is calculated using these models after the effects of aging have been added. The temperature (T), depth of discharge (DOD), state of charge (SOC), and discharge velocity (C-rate) are the primary variables that influence battery aging mechanisms [49,50,51].

Analyzing the aging phenomenon is necessary to keep track of a battery’s useful life. Batteries experience both calendar aging, which occurs when they are stationary, and cycling aging, which occurs when they undergo cyclic operation [35]. The temperature and the SOC are two primary factors that contribute to aging in the first scenario, in addition to time itself. The Arrhenius equation can be used to explain how the temperature changes exponentially [52], whereas the SOC changes linearly [53,54]. On the other hand, the DOD and the C-rate also play a role in aging brought on by cycling [55]. In contrast to the C-rate, which uses a second-degree polynomial, the first one accomplishes this using a logarithmic relation [56,57].

Both a rise in the battery’s internal resistance and a reduction in capacity are the two consequences that these effects have in practice [58]. The RC components are also affected, but these effects only have an immediate impact on how quickly the battery reacts to sudden changes in current. Practically speaking, a relationship can be drawn between the battery’s capacity loss and aging, which will continue to happen continuously despite taking place slowly.

The gradual loss of a battery’s capacity and its increase in internal resistance are the most obvious effects of battery aging, and these are measured by the state of the battery (SOH) parameter. The SOH can be calculated either using Equation (1) or (2) as the ratio of the battery’s current capacity (Cap) to its initial capacity (Cap_ini_) or its initial internal resistance Rn_ini_ to increased internal resistance Rinc, respectively.

SOH = Cap/Cap_ini_ (1)

SOH = Rinc/Rn_ini_ (2)

Its operational limit will be established by the SOH. In other words, this is the parameter that determines when a battery in a given application reaches the end of its useful life. The aging of batteries and other relevant parameters have been analyzed through experimental tests performed in laboratories following predetermined protocols [59,60,61,62,63].

When taking into account a battery’s actual operating circumstances, sensors may frequently be unable to determine or estimate aging parameters by measuring their internal variables. Mathematical models developed in experimental laboratory conditions do not easily adapt to the random conditions of complex degradation associated with the real-world operating regime that batteries are subjected to in challenging and difficult circumstances because measurements can be very challenging, expensive, or intractable. Batteries gradually degrade under these circumstances as a result of multi-parametric cumulative effects, the relative contributions of which are very difficult to measure. Online trend analysis techniques are more suitable for monitoring the true state of charge (SOH) of batteries when operating under actual conditions [64].

### 1.4. Common Catastrophic Battery Failures

Finding out when batteries’ useful lives are up with the potential to move on to adequate maintenance in the appropriate time and form is one of the key components of diagnosis. However, it is also important to take into account the risks related to some common catastrophic failures, whose effects might be felt immediately. According to their technology, batteries are known to fail in a variety of ways [65]. Due to their relative advantages, relatively low cost of installation and maintenance, energy density, and safety, some of the most popular battery types today are the VRLA and gel types. The associated typical failure modes for these types of batteries are those that are listed below [66,67]:

- Dry-out (loss-of-compression);
- Plate sulfation;
- Soft and hard shorts;
- Post leakage;
- Thermal runaway;
- Positive-grid corrosion.

Many of the above-mentioned failure modes, particularly dry-out, positive-grid corrosion, and thermal runaway, are strongly influenced by an increase in the internal battery temperature, which in turn depends, under normal circumstances, primarily but not exclusively on the ambient temperature resulting from environmental factors such as weather. The battery installation chamber acts as a “filter” to change the outside temperature to the ambient temperature.

The internal battery temperature has a significant impact on aging, grid corrosion, and the rate of water loss (dry-out) from evaporation or hydrogen evolution at the negative plates (self-discharge), all of which rise with temperature. On the other hand, applications that involve intense cycling may benefit from a (moderate) temperature increase [68].

The phenomenon of dry-out occurs and is accelerated by excessive heat (lack of proper ventilation or, to put it another way, heat accumulation inside the battery as a result of a prior failure from the heat dissipation process), as well as overcharging, which can result in elevated internal temperatures and high ambient (installation chamber) temperatures, and significantly contributes to grid corrosion. Failures can show signs of drying out in between 82 and 85% of cases [67]. It frequently occurs as a side effect of some failure modes and as a unique inducer of others, such as thermal runaway. Negative-strap corrosion, which causes the slow loss of the electrolyte, is the typical failure mode for a VRLA battery under normal operating conditions. The pressure relief valve (PRV) allows the sealed cells to vent when the internal temperature rises. The battery capacity decreases, and the internal impedance rises when enough electrolyte is vented, removing the glass matte from contact with the plates.

When a battery experiences thermal runaway, its temperature rises quickly, causing it to overheat to an extreme degree. As a result, the battery may melt, catch fire, or even explode. Only high ambient temperatures and/or excessive charging voltage can cause thermal runaway in a battery [69,70]. Even though runaway failures are less common, they can still have critical consequences. In these situations, the battery system’s automatic control action must be immediate and based on its quick isolation from the loads and disconnection from the generation sources, both of which must be carried out by the BMS system as a whole. In a self-sustaining reaction, thermal runaway happens when a battery’s internal parts start to melt. The battery’s internal temperature increases as the current is accepted. The battery can accept more current from the charger because the temperature increase lowers the impedance of the battery. The battery gets hotter as a result of the higher current. When the heat removed linearly increases more slowly than the heat produced by the reaction, thermal runaway occurs. As a result of the reaction mass’s temperature being raised by the excess heat, the rate of reaction rises. In turn, this quickens the rate at which heat is produced. According to a rough rule of thumb, the reaction rate—and subsequently, the rate of heat generation—doubles with every 10 C increase in temperature, causing the battery temperature to “runaway”. The temperature can rise even further to the point of plastic meltdown and potential fire [66] once the electrolyte has boiled away, exceeding the upper limit of 126 °C [67] that will eventually be reached when it begins to boil.

The float voltage and ambient temperature can have varying effects on various batteries. Significant variations in the float voltages between various batteries of various makes and models can result in varying aging times. The chemistry of the battery and its construction, its age, and particularly the chamber conditions where batteries are installed all play a part in the response variation in each battery’s behavior [71].

If the battery’s internal heat generation process enters an advanced uncontrolled phase, violent boiling and quick gas generation will take place, leading to over-pressurization. If this condition is not caught in time, it can result in catastrophic damage due to emissions of hydrogen, oxygen, hydrogen sulfide gas (an irritant), and atomized electrolyte. The installation chamber may catch fire or explode as a result of this process [72]. Users may be exposed to hazardous gas emissions in this situation, including hot, toxic gases, liquids, and particles, increasing the possibility of catastrophic mishaps.

All batteries are known to be “killed” by high temperatures, though the impact varies depending on the model, manufacturer, and manufacturing technology used. At 95 °F (35 °C), the life of lead–acid is reduced by 50%, whereas the life of nickel–cadmium is reduced by 16–18% [56,62]. The battery life is halved for every 18 °F (10 °C) increase in battery temperature. Positive-grid corrosion occurs more quickly as a result of the elevated temperature, as do other failure modes. A 20-year battery will only last 10 years if it is kept at a temperature of 95 °F (35 °C) as opposed to the intended 77 °F (25 °C), and so on. A 20-year battery will only last 5 years if the temperature is raised by another 18 °F to 113 °F (45 °C). The internal temperature of the batteries is therefore the most crucial parameter to take into account in predictive trend analysis [56].

On the other hand, any battery’s internal chemical reactions are slowed down by the low-temperature range. Depending on the technology, the degree of performance reduction varies as well. A VRLA battery might need a 20% capacity compensation, for instance, when the temperature is close to freezing. A capacity increase of twofold is necessary for a lead–calcium cell using 1.215 specific gravity acid, compared to an 18% increase for the Ni-Cd cell.

In a perfect world, the trend analysis of some battery parameters, especially temperature, impedance, capacity, and their relationship with SOH, would be a great tool for determining when it is time to replace the batteries and how the batteries degrade over time. However, as was already mentioned, not all of these parameters can be easily assessed [73,74,75].

However, despite the unquestionable advancements brought about by the introduction of BMSs, there are still some issues that limit the ability to fully utilize the potential for performing estimations with more precise models referring to the internal state parameters of the batteries, such as SOC and SOH. The limitations on computing power and data storage that are currently in place make this problem more difficult. The most recent proposals that have been made in recent years, based on the use of IoT, cloud computing, twin models, big data, and machine learning, aim to eliminate the current difficulties [76,77,78,79].

In this work, as a contribution, a decentralized but synchronized real-world smart battery management system has been designed using a Cerbo GX general controller with networking communication capability and cloud data processing access, four charge regulators, and a sensorized smart battery monitor with networking and Bluetooth capabilities. Currently, BMSs can be utilized as distributed control systems for real-world applications when general controllers, charge regulators, and smart monitors and sensors are integrated, such as those suggested in this work, which enable more accurate estimations of the battery’s electrical parameters.

The main feature of the proposed BMS is that it is intelligent, as it provides the ability to supply dynamic parameters from a non-intelligent battery in a similar way to intelligent batteries. It is a real-world BMS system and a comparatively low-cost system.

Another contribution made in this work is the development of a methodical modeling procedure based on Petri Nets, which establishes, in a visible, organized, and precise way, the set of conditions that will determine the operation of the BMS. If this modeling is not carried out, the threshold values and their conditions remain scattered, not very transparent, and difficult to deal with in an aggregate manner.

This document is structured as follows: In Section 2, the smart devices and sensors used to monitor an energy storage system (ESS) are presented. The tests conducted are described in detail, and a decentralized but synchronized real-world system for a smart BMS system is proposed. The suggested management approach is predicated on the articulation of particular parametric conditions based on the type of batteries employed, whose dynamics can be represented as Petri Nets models (PNs). The experiment’s results are presented in Section 3. Section 4 discusses the findings as well as some additional suggestions and information gleaned from the experimental trials. Finally, in Section 5, some inferences are made regarding the benefits of the suggested approach to managing batteries.

## 2. Materials and Methods

### 2.1. Materials

**Figure 1:**
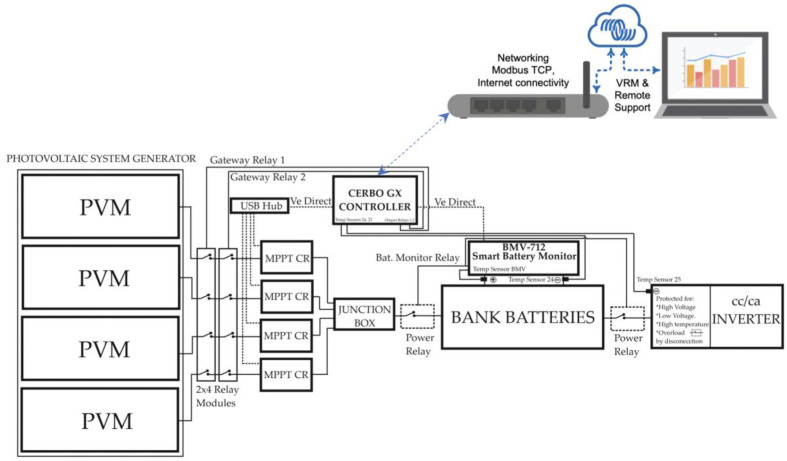
Photovoltaic generation system with integrated and distributed smart BMS system.

This section shows the materials used to configure the BMS system. Figure 1 shows the representative block diagram of the integrated intelligent BMS. Every block is explained in the following paragraphs.

#### 2.1.1. Batteries

**Figure 2:**
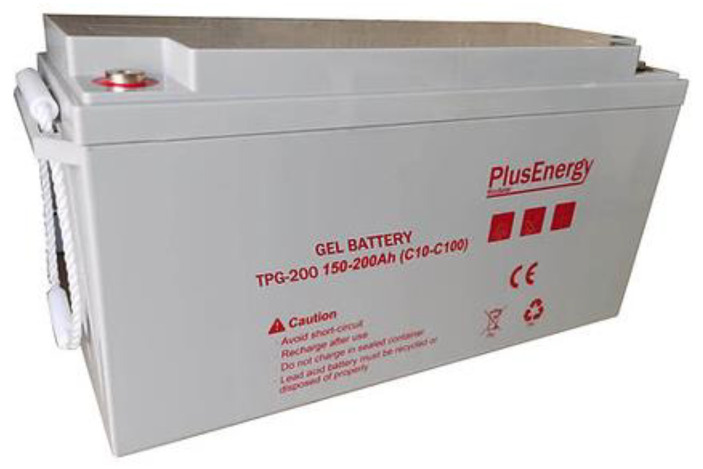
PlusEnergy Gel Battery TPG 12 V 150–200 Ah (C10–C100).

The PlusEnergy TPG200 GEL Battery is a so-called maintenance-free type, as it is a sealed monoblock battery that can be installed in indoor locations with poor ventilation (Figure 2). It is made of ABM and is highly resistant to acid and demanding external conditions. Although it undergoes deep discharges quickly, it is recommended that its discharges not be greater than 30%, since otherwise, it can significantly shorten its useful life.

The technical data of the battery are as follows: voltage: 12 V; model: TPG12-200; dimensions: 483 × 170 × 170 × 240 × 240 MM; weight: 41.5 Kg; brand: PlusEnergy; battery type: GEL; maintenance-free period: more than 3000 cycles according to IEC 61427; capacity: 200 Ah in C100 and 150 Ah in C10; 2-year warranty; battery applications: telecommunications, repeaters, solar installations, batteries for use in enclosed areas; fields of application: security system, backup power supply, UPS, new energy storage, medical device, electric toy, communication system, computer system, emergency light, solar system, wind power system.

**Figure 3:**
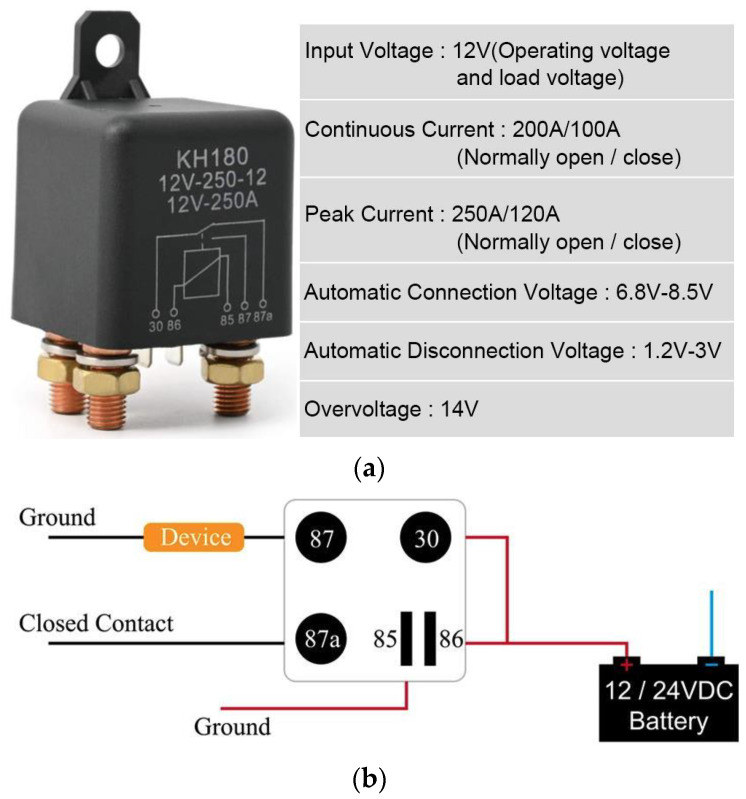
Battery relay: (**a**) relay properties; (**b**) connection to the battery.

Figure 3 shows the connection and disconnection power relays used for this battery.

#### 2.1.2. Xuncel Inverter

**Figure 4:**
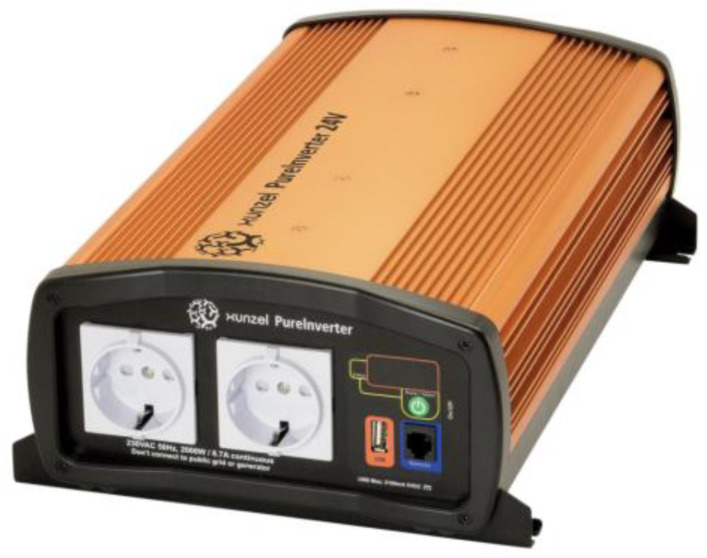
Inverter XJC20001255U Model.

The XJTM XUNZEL Series pure sine wave inverter is an inverter that changes the DC input voltage of a battery to a symmetrical AC output voltage (Figure 4). The output waveform is a high-quality pure sine wave with total harmonic distortion below 3% (THD < 3%).

#### 2.1.3. BlueSolar MPPT Regulator

**Figure 5:**
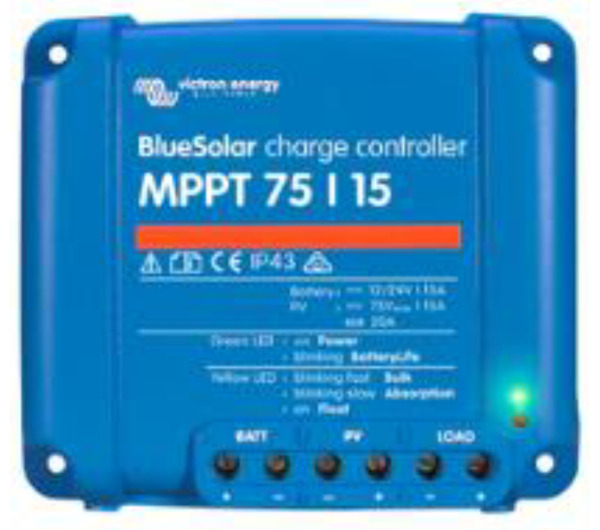
Victron MPPT Solar Controller.

**Figure 6:**
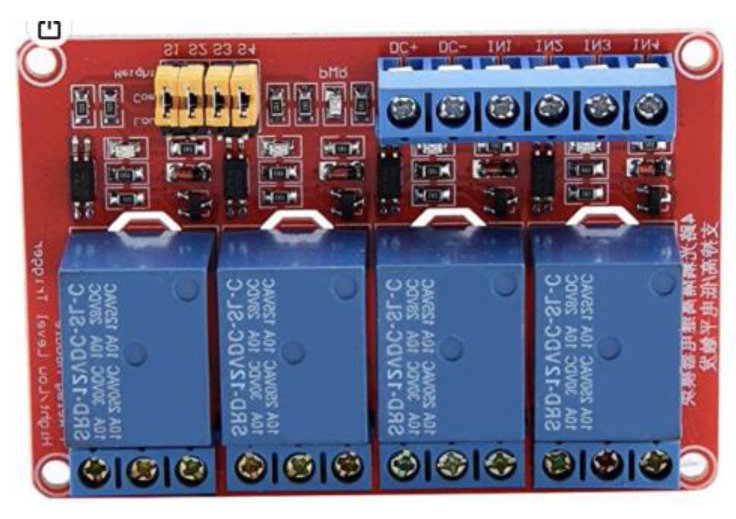
Photovoltaic generation system: 4-relay modules of on/off actuator.

One of the primary purposes of charge regulators is to continuously monitor the battery’s condition and control the intensity of the charge in order to increase the amount of electrical energy that can be generated from energy sources. Depending on the battery charge level, if it is lower than 95 percent, it will allow all of the energy produced by the solar panels to pass through so that they can be charged as quickly as possible. The energy flow will be extremely controlled to allow for maximum charging if the batteries are between 95% and 99% charged (the float state). The power supply will be cut off if the batteries are fully charged, protecting them from overheating and overloading. As a result, the initial battery bank’s SOC will determine the amount of charge that the solar regulators must distribute. The battery will always be safeguarded against overloads thanks to the solar charge controller (SCC), and the charge is executed whenever it is most practical (Figure 5). Figure 6 shows the connection and disconnection relay module.

**Figure 7:**
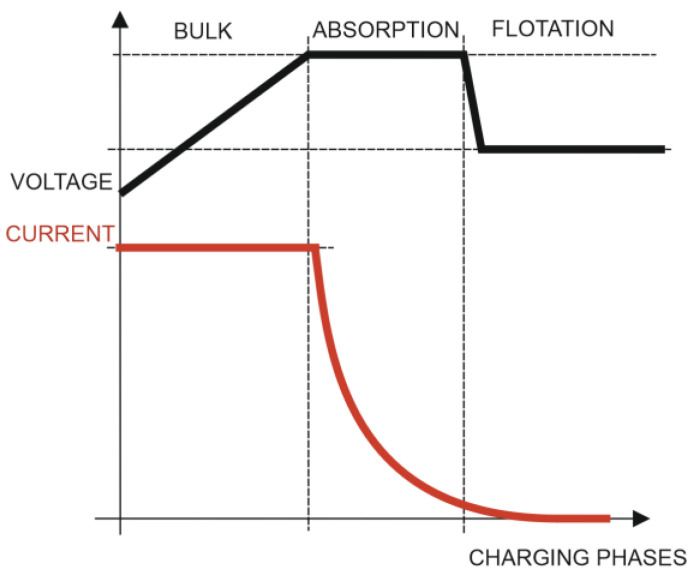
Batteries’ three-phase charge process.

The three charging phases used by the solar charger are as follows (Figure 7):

- Initial charge (bulk): The solar charger uses its maximum charge current during the initial charge phase to quickly charge the batteries. The battery voltage will gradually rise during this stage. The initial charging phase of the battery ends when the voltage reaches the predetermined absorption voltage, and the absorption phase then starts.
- Absorption: The solar charger is now operating in constant voltage mode as it enters the absorption phase. The battery’s current will gradually drop. The absorption phase ends and the float phase starts when the current drops below 1 A (tail current). The absorption time is brief when there are only surface discharges. This stops the battery from being overcharged. The absorption time is automatically extended to ensure that the battery is fully charged following a deep discharge.
- Float: The batteries are fully charged during the float phase when the voltage is decreased.

The VictronConnect app allows one to select from eight preset charging algorithms. It is also possible to fully program the charging algorithm. It is possible to adjust the charging voltages, the length of the phases, and the charging current.

Periodic charging for equalization is necessary for some lead–acid battery types. The charging voltage will increase during equalization to balance the cells above standard charging voltages. Applications such as VictronConnect can enable equalization charging if it is necessary.

Because the battery temperature affects the absorption and float charge voltages, a specific temperature sensor is necessary. The settings for the solar charger allow one to enable or disable temperature compensation. By using the compensation coefficient (mV/°C), the compensation amount can be changed.

When greater accuracy is needed, using an external battery temperature sensor should be taken into consideration. The temperature compensation range is 6 °C to 40 °C (39 °F to 104 °F). The internal temperature sensor of the solar controller is also used to determine whether the solar charger has overheated. In order to improve the charging efficiency and lengthen the life of lead–acid batteries, it is ideal to take into account a wireless battery voltage, current, and temperature sensor that can be used in conjunction with the solar charger. Additionally, it compensates for any voltage drops across the battery cables that may occur when charging with a high current by increasing the charging voltage.

#### 2.1.4. BMV-712 Smart Battery Monitor

**Figure 8:**
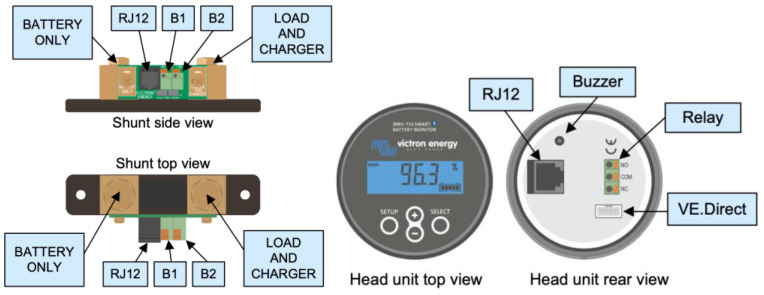
BMV-712 smart device.

**Figure 9:**
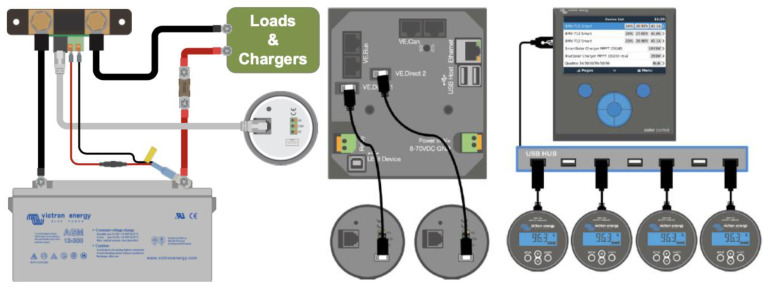
BMV-712 smart device installation.

The smart device used in this work is the BMV-712 (Battery Monitoring Victron) Smart Battery Monitor (Figure 8), which can be connected to the general Cerbo GX controller via a VE.Direct port (Figure 9). It can also be connected with the VE.Direct interface via USB and wirelessly, so it can work synchronously with the solar charger. Thus, it is able to send it the temperature and voltage values of the battery to perform the best possible charge compensation. In addition to local monitoring and control with the Cerbo GX, the information is also sent to the free remote monitoring website: The Victron Remote Monitoring (VRM) Online Portal.

The main function of the BMV-712 is to monitor and indicate the state of charge of the battery by monitoring the current flow into or out of the battery, preventing an out-of-control total discharge from happening. A simple way to calculate the charge would be by integrating this current over time (which, if the current is a fixed number of amps, reduces to multiplying the current by the time). This operation will give us the net amount of Ah charged or discharged. Unfortunately, however, the calculation of the effective capacity of a battery is particularly complex since its dependence is multifactorial, since (a) the discharge process is conditioned by the speed at which it is produced and (b) also depends on the temperature at which the discharge is carried out, and (c) another factor that increases the complexity is produced during the battery charging process due to the occurrence of the phenomenon known as gassing [3]. Due to this phenomenon, there is a loss of efficiency in the charging process, since the total Ah supplied for charging experiences a decrease in the final amount of Ah stored in the battery.

##### External Temperature Sensor

**Figure 10:**
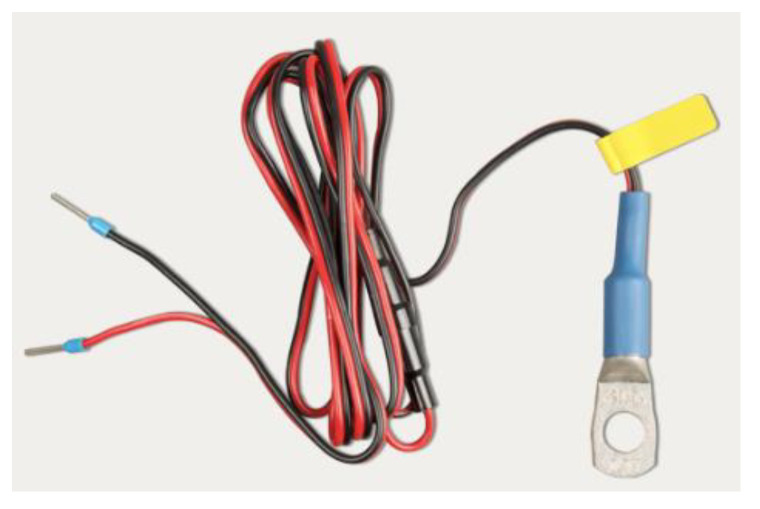
ASS000100000 temperature sensor.

In order for the BMV-712 Smart Battery Monitor to incorporate more accurate temperature measurement values, it must incorporate a compatible temperature sensor, such as ASS000100000. The temperature sensor should be connected between the shunt connectors and the positive terminal of the battery bank, as shown in Figure 10, where one of the two wires of the sensor functions as a power cable. The temperature can be displayed in °C/°F, and its measurement can be used to adjust the battery capacity to the temperature.

The monitor displays and sends to the network, among others, the following parameters:

- Battery voltage;
- Voltage of the auxiliary battery;
- Input current (without sign)/output current (with sign -);
- Power drawn (sign -)/power added (unsigned);
- Ampere hours consumed: Ah quantity (with sign -);
- SOC (0–100%);
- Remaining autonomy (h);
- Battery temperature (°C/°F).

Battery charging with temperature compensation is very convenient, especially when charging batteries in environments subjected to hot or cold climates. This compensation can be enabled or disabled during the configuration phase of the solar charger and in the BMV-712 device. With this setting, the amount of compensation can be adjusted by assigning a compensation coefficient (mV/°C).

#### 2.1.5. Cerbo GX General Control Device

**Figure 11:**
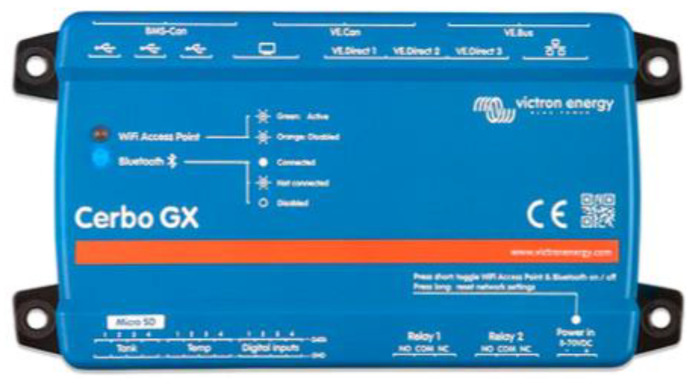
Cerbo GX Controller.

In the context of the configuration of a decentralized intelligent battery management system, the Cerbo GX device will be used as an overall system controller with the ability to communicate with the other devices both wirelessly and directly through VE.Direct ports (Figure 11). Both ways ensure communication with the solar chargers, BMS, and inverters.

The Cerbo GX, which ensures the coordinated operation of the entire installation, is connected to the other parts of the system, including the solar regulators, inverters/chargers, surveillance and monitoring devices, and batteries.

##### Victron Connect-Remote App

**Figure 12:**
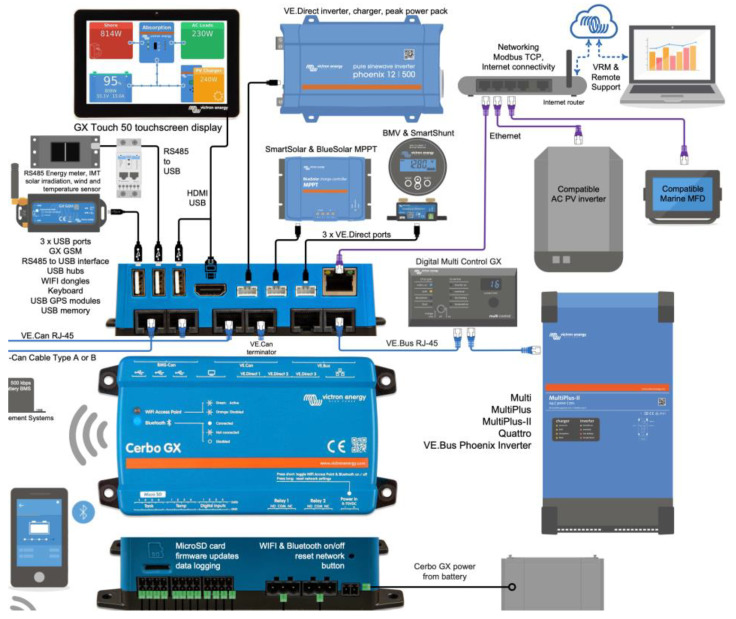
Cerbo GX cloud networking devices configuration.

Victron Connect-Remote allows remote access through the online VRM portal by communicating with a general controller, such as the Cerbo GX, which enables a wide variety of Victron devices with a VE.Direct interface. This powerful option allows monitoring, operation, and configuration with complete system monitoring from virtually anywhere in the world. With this user interface, the perception can be experienced as if the BMS system were connected locally via Bluetooth or wired to VE.Direct via the USB interface. Monitored online data such as power, voltage, current, temperature, and other information are constantly transferred to a big data platform based on cloud technology with massive data storage and processing potential (Figure 12).

The multiple models will increase adaptation to uncertain environments and different aging levels, so a more accurate and reliable estimation of SOC can be achieved under complicated operating conditions.

##### Cerbo GX General Control Device with Temperature Sensors

**Figure 13:**
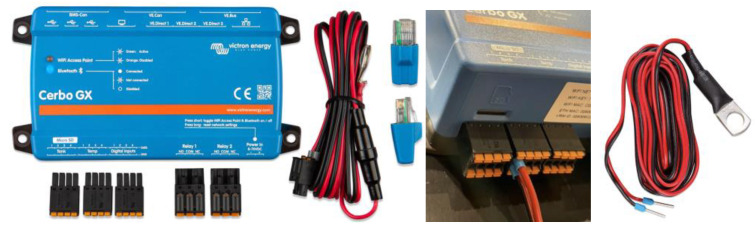
Cerbo GX, temperature sensor inputs, and relay output.

The Cerbo GX controller also has 2 additional relays (Figure 13) with the ability to act on the system not only to bring the battery to a safe state but also to perform control actions leading to the start/stop of generators and inverters. These parameters, such as the SOC parameter, voltage, current, and especially temperature, can be obtained more accurately from the BMV-712 intelligent device in order to extend the life of the batteries by keeping them at adequate safety values.

With this in mind, the Cerbo GX can be connected to a set of up to 4 Ruuvi wireless temperature sensors or ASS00001000 wired temperature sensors. These sensors can be used to control the start/stop actions of generators and inverters, ensuring battery safety in cases of dangerous temperature rises in other devices such as inverters, battery installation chambers, and cold rooms.

### 2.2. Methods

The designed method of battery parameter status management is based on the use of a distributed battery management system. The precise measurement of the SOC, V, I, and T parameters of the battery are performed specifically by the BMV-712 intelligent device, as well as alarm processing. The actuation response of the safeguard system can be split between the BMV-712 itself, which has an integrated relay, and the general controller of the Cerbo GX system, which, as shown above, has two additional integrated relays.

The advantages of the decentralization or distribution of control tasks began to be recognized as early as the 1970s when control systems were centralized by a single computer due to their high costs. The failure of a single centralized computer meant the shutdown of the entire controlled system. Currently, the implementation models of automated systems are based on the use of distributed (decentralized) control systems so that a failure of a decentralized controller will only locally affect a specific part of the process. In the designed distributed system, four MPPT charge controllers are used. Should one of them fail, the power supply will not be interrupted since the others will be able to supply the necessary charge, protecting the batteries and preventing them from experiencing a significant level of discharge. However, the solar chargers used, although they are independent, must operate synchronously when it comes to developing the bulk, absorption, and flotation charging phases.

The synchronization of the chargers is carried out through a type of master–slave system algorithm used by the Cerbo GX general controller. The algorithm chooses a master among the set of connected solar chargers in the system, and that master will be the one that dictates the charging algorithm; however, all the solar chargers that belong to the same network must be configured for the same type of batteries by the programmer. Then, the master will make sure that all chargers are in the same state of charge and at the same voltage setpoint. At the start of the day, the master will measure the battery voltage before any of the other chargers in the network start charging (to find the idle voltage of the battery). This information is used to decide what the total absorption time should be for some types of batteries. The idle voltage of the battery, as well as the total absorption time and the time spent in the current state of charge, is shared with the other chargers. That information is important so that chargers can resume the charging algorithm if, for some reason, the master stops charging (i.e., the Sun went down or solar energy hitting its panels decreased, the charger went out, the charger lost contact with the grid, etc.).

To ensure the synchronization of the charge controllers, a Cerbo GX controller is used in conjunction with four identically configured SmartSolar charge controllers. One possible configuration is to use a VE.Smart network to charge the battery banks, fulfilling the same mission as a single higher-power regulator. The decentralized solar chargers will be synchronized using the same charging algorithm between them, without the need for additional hardware, performing the bulk, absorption, and float charge state changes synchronously. Each charge regulator has the ability to manage its own output current, which will depend mainly on the output of each photovoltaic solar panel, the resistance of the cable, and the maximum output current configured in the charger, but sometimes it is convenient to configure the maximum load current of the entire network. In case this function is needed, the distributed current and voltage control functions (DVCC) must be used; for this objective, in this work, a Cerbo GX device was used.

Gel batteries were used in this work, since their use is frequent in many solar installations due to their comparatively low cost compared to lithium batteries. In gel batteries, it is advisable to confine the SOC to 80–100% to ensure the best possible lifetime. The battery management system’s measurement of the SOC to reduce extended periods of SOC decline is one of its primary goals. Because of the seasonal decrease in solar radiation during the winter, the battery may experience a low SOC for protracted periods of time. This will result in an increase in the sulfation phenomenon and significantly shorten the battery’s lifespan. Because battery self-discharge starts to play a significant role in long-term energy storage, the battery is unable to offer enough storage capacity for the worst season of decreased solar radiation [11]. In this regard, the use of controllers with maximum power point tracking (MPPT) is advisable, as it adapts the operating voltage in the PV system to provide the maximum power, managing to increase the solar panel voltage and increasing the solar production by up to 30%. Otherwise, the battery life may be shortened by overcharging due to the use of an inaccurate charge regulation system. To prevent overloads in a precise way, it is necessary to ensure the synchronized operation of the set of devices that are part of the generation system, as well as its charge regulation system.

Therefore, to ensure the confinement of SOC values in a given operation range, the synchronized operation of the set of solar charge regulators that are part of the intelligent battery management system via distributed voltage and current control (DVCC) functions has been used.

#### 2.2.1. Distributed Voltage and Current Control (DVCC) Functions

**Figure 14:**
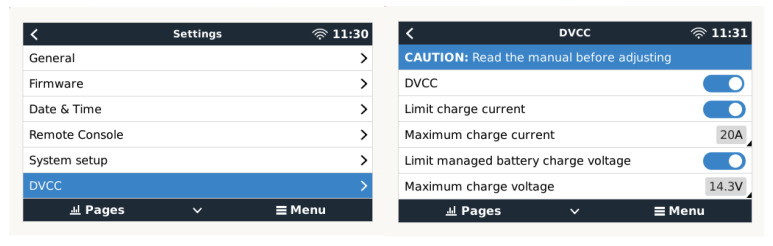
Distributed voltage and current control functions (DVCC).

The bulk, absorption, and float charge state changes will be carried out concurrently by the decentralized solar chargers using the same charging algorithm. Each charge regulator has the ability to control its own output current, which is determined by the output of each solar panel, the cable’s resistance, and the maximum output current set by the charger regulator’s maximum charge-current-limiting function for the network as a whole. In this case, this function is associated with the distributed voltage and current control (DVCC) functions [80] of a Cerbo GX software device (Figure 14).

In systems with lead–acid, AGM, and GEL batteries, but also Li-ion and LiFePO4 batteries, the DVCC functions allow configurable charging current limits to be set for the entire system, where the Cerbo GX device actively limits the inverter/charger in case the solar chargers are already charging at full power. Additionally, it allows the provision of more accurate values, such as those provided by the shared temperature sensor (STS), current sensor (SCS), and voltage sensor (SVS), which can be obtained from the BVM-712 smart device connected to a VE.Direct port of the Cerbo GX controller [80]. These functions are described in more detail below:

- The SVS is enabled when DVCC is enabled. It works with solar chargers connected to a VE.Direct port. The system automatically selects the best available voltage measurement obtained from the BMV-712 battery monitor. To make up for the voltage loss in the battery wires, the battery voltage data are used. This prevents a lower voltage from charging the battery than the exact voltage set on the charger owing to wiring resistance.
- The STS will use the BMV-712 to obtain the battery temperature value for the Cerbo GX controller device to send the obtained temperature measurement to the inverter/charger system and all connected solar chargers. The battery temperature data are used to adjust the charging voltages. At low temperatures, a lead–acid battery generally needs a higher charging voltage, and conversely, a lower charging voltage is required at high temperatures. When using lithium batteries, the charging voltages are the same at all temperatures, except at low temperatures. Below 5 °C, they should not be charged to prevent damage and degradation; therefore, they should be disconnected from the generation source.
- The SCS sends the battery current, measured by the BMV-712 battery monitor connected to the GX device, to all connected solar chargers. The battery current data are used to enable more precise usage of the tail current setting because it helps the solar charger determine when the absorption phase should end and transition to the equalization/float phase. However, it should be noted that gel batteries such as those used in this work are generally not suitable for the equalization phase.

#### 2.2.2. Alarm, Acoustic Signals, and Relay Management

For most of the readings of the BMV-712 smart device, an alarm can be configured that will be triggered when a preset threshold value is reached. When the alarm is triggered, a beep will sound, the backlight will flash, and the alarm icon will appear on the display along with the current value. When an alarm occurs, an associated relay built into the body of the monitor can be optionally triggered as a reactive actuation on the system in response to the alarm activation. The alarm is canceled by pressing a button. However, the alarm icon will continue to be displayed as long as the alarm conditions remain in effect.

The BMV-712 relay is installed in the battery monitoring device itself and will be activated/deactivated when the threshold values of the parameters listed in the left column of Table 1, all battery-specific parametric values [11], are reached. In the right column of Table 1 are the BMV-712 parameters that are activated/deactivated in alarm management and processing but do not give rise to any relay trips.

#### 2.2.3. Dynamic Model of System Operation

A dynamic system controlled by discrete events was used to model the management of the safe operation of the ESS of a battery bank, which is specifically based on a Petri Net (PN) model, which, due to its dynamic characteristics, allows the intuitive representation of the set of precise actions to be performed on the system depending on the realization of a series of threshold values of characteristic parameters taken from the specific characteristics of both the batteries and the inverter. See Supplementary File.

**Figure 15:**
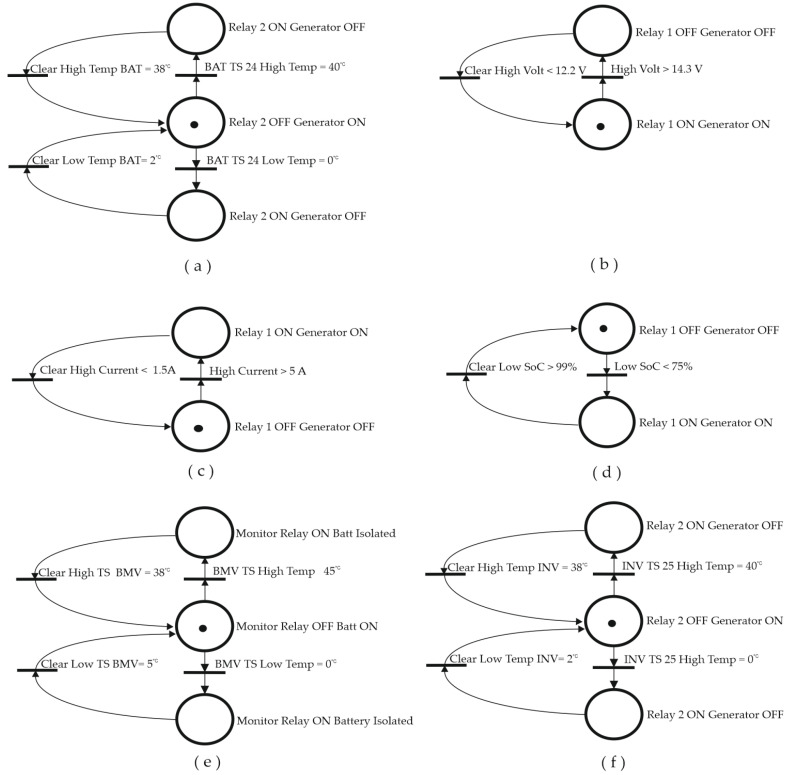
(**a**) Start/stop generator by Temperature Sensor 24 PN Model. (**b**) Start/stop generator by Voltage PN Model. (**c**) Start/stop generator by Current PN Model. (**d**) Start/stop generator by SOC PN Model. (**e**) Connection/disconnection of battery by BMV Temperature Sensor PN Model. (**f**) Start/stop generator by Temperature Sensor 25 PN Model.

The design, configuration, and implementation of the algorithm to be programmed based on conditions will allow the establishment of the set of triggering conditions of PN transitions, and the corresponding actions to be performed on the system will be assigned to the corresponding output location. Figure 15 shows the PNs corresponding to the treatment of the parameters temperature, voltage, current, and SOC.

## 3. Results

These results describe the application of the set of BMS functions used in dedicated tests for the start/stop of the photovoltaic generation system conditioned by the following parameters:

- Battery voltage;
- Battery SOC;
- Battery current;
- Battery and inverter temperature.

For the specific case of the work developed in our study, the following configurations were arranged based on the conditions of the system used.

### 3.1. Battery Charging

**Figure 16:**
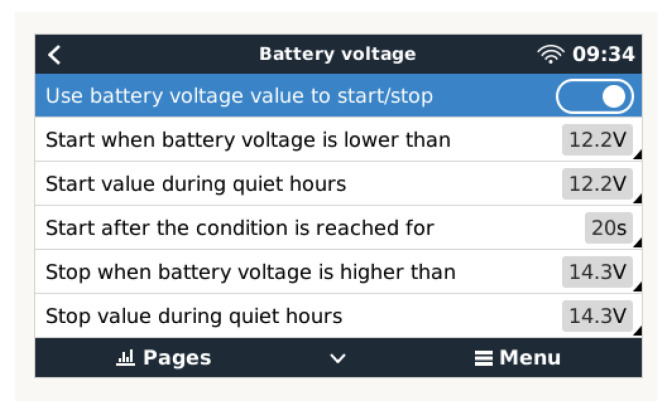
Condition configuration based on voltage.

In the configuration shown in Figure 16, the conditions based on battery voltage thresholds are established, performing charging from the voltage drop to 12.5 V, allowing the batteries to be charged following the bulk, absorption, and flotation phases. The BMV is reset to “fully charged” in the case of a 12 V battery when the following “charging parameters” are satisfied: the voltage exceeds 13.2 V while the charging (tail) current is less than 4.0 percent of the entire battery capacity (i.e., 8 A in a 200 Ah battery) for three minutes.

### 3.2. Supplementary Charge Conditioned by Current Consumption

**Figure 17:**
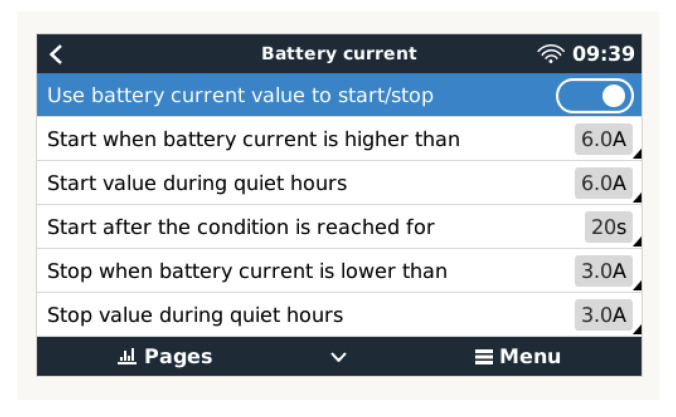
Condition configuration based on current.

This condition is used outside the normal charging phase sequence that results in reaching the 100% SOC level. Once this condition is reached, the generator will be automatically disconnected. However, if, in this generation system shutdown phase, an increase in battery current consumption occurs, such as the one set in the following window, it will allow the automatic restart of the generation system to prevent the premature discharge of the battery. In the configuration shown in Figure 17, any consumption above 6 A will trigger the restart of the generation system, which will be suspended when the load consumption drops below 3 A.

**Figure 18:**
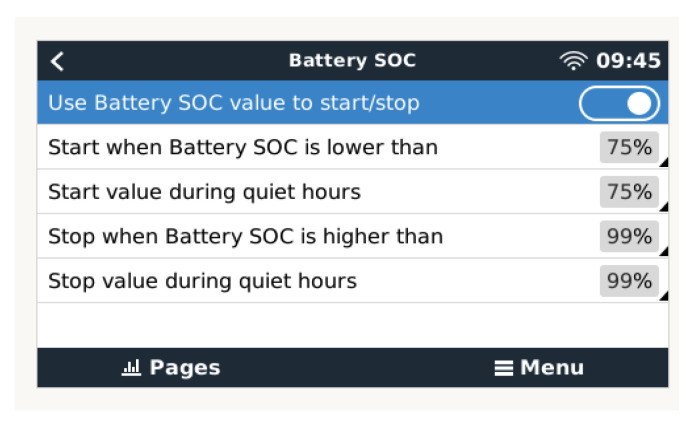
Condition configuration based on SOC.

Finally, reaching 100% SOC will cause the generation system to stop until a new charging operation is required due to consumption that results in a drop to 75% SOC (Figure 18).

Table 2 and Table 3 show data obtained from the cloud server corresponding to the application of the generator start/stop control based on the condition of the voltage, current, and SOC parameters. Table 2 shows data corresponding to the beginning of the morning, when the BMS triggers a low-voltage alarm due to the energy consumption occurring during the night. The columns with the states of the four relay modules connected in series used for the start/stop control of the four solar controllers are shown. The Generator run reason column shows the currently active condition, as well as the activation of the low-voltage alarm status. In this situation, Relay 1 (NO) must be activated (closed), allowing the start of the solar generation system. Relay 2 remains inactivated, but it has been configured in a normally closed (NC) situation.

In Table 3, a wider variety of conditions are shown, such as stopping the solar generation system due to high voltage values, as well as additional current charge supplies when peak load consumption occurs to prevent the battery from discharging prematurely. These conditions are automatically established during data processing in the cloud. In column D of Table 2 and Table 3, they are defined as the Generator run reason; that is, the conditions of the electrical parameters that cause the alarms are established.

**Figure 19:**
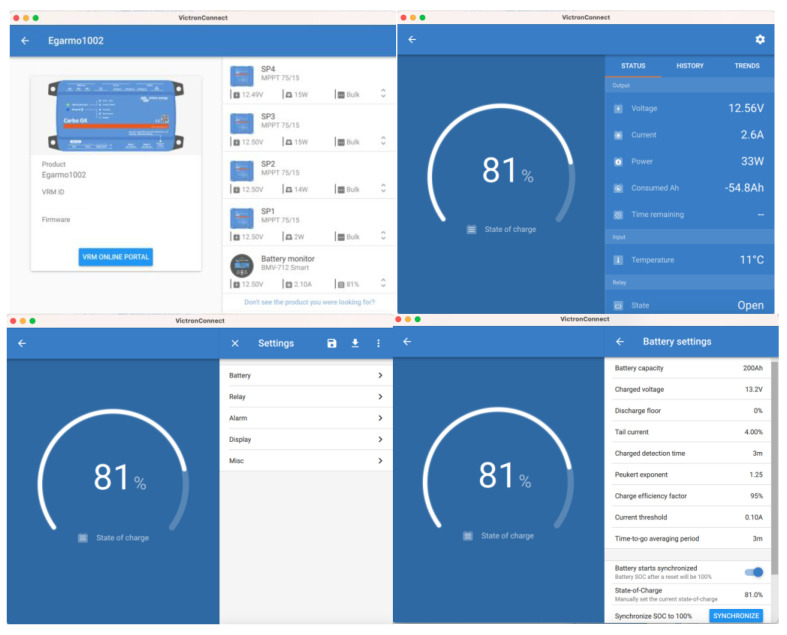
Optional user synchronization to 100% SOC.

The option of using charge management based on the configuration based on the conditions of the electrical parameters V, I, and SOC is appropriate in the case of generic charge generators. However, in the case that the generation is carried out with solar chargers, it is more appropriate that the charging algorithm is directly managed by the advanced charging algorithms of the MPPT type, such as those used in this work. The three charging phases used in this case by the solar charger are:

- Initial charge: In the first (bulk) phase of charging, all available current from the solar charger is used to charge the battery to the absorption voltage of 14.4 V. In this phase, a gel battery is charged to about 85%.
- Absorption: During this phase, an absorption voltage of 14.4 V is maintained for a variable time that is set by the solar charger algorithm, taking into account the information supplied by the BMS-712 device regarding the initial state of charge of the battery, and can be up to 5 times longer than the duration of the bulk phase. The charging current is gradually reduced until the float current is reached.
- Float: Once the absorption phase is finished, after a time that will depend on the battery charging algorithm selected in the MPPT solar charge controller, the flotation phase is reached. At this moment, the battery is considered 100% charged. In the case of a 12 V battery, the BMV resets to “fully charged” when the following “charging parameters” are met: the voltage exceeds 13.2 V, and simultaneously, the charging (tail) current is lowered to 4.0% of the full battery capacity. In the case of the type of 200 Ah battery used in this work, it is 8 A for 3 min. Once this state of charge is reached, the VictronConnect application executes automatic synchronization, reaching 100% SOC. Synchronization can also be optionally executed by the user using the configuration sequence shown in Figure 19. Once the first synchronization is completed (automatically or manually), the BMV keeps a record of the automatic synchronizations that occur (Figure 19).

### 3.3. Test Management on High/Low-Temperature Alarm Threshold Values

**Figure 20:**
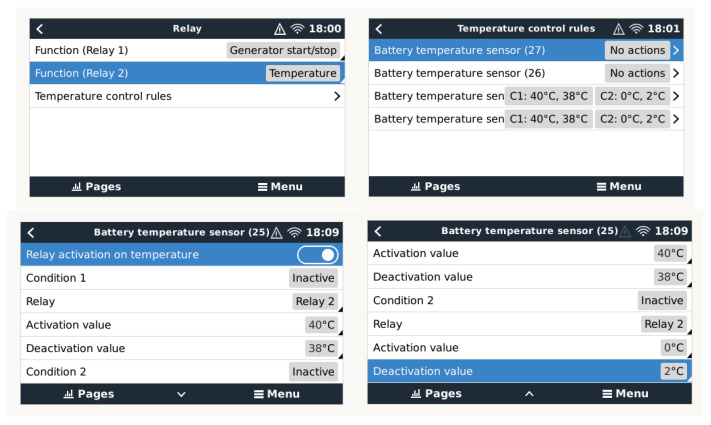
Condition configuration based on Temperature Sensors 24 and 25 and Cerbo GX Relay 2.

To carry out the safe management of high/low-temperature threshold value alarms that may eventually affect batteries or inverters, the generator must be stopped/started. For this purpose, the configuration sequence that must be used is shown in Figure 20. In our tests, we used two temperature sensors, 24 and 25, acting on the same Relay 2 to manage the start/stop of the generator, since Relay 1 is dedicated by default to the start/stop management of the generation system due to the V, I, and SOC parameters.

In order to carry out these tests, destructive testing was avoided, taking into account the dangerous nature of the tests. Thus, an inductive method was used to introduce threshold temperature limit values through the application of high and low temperatures by applying a Peltier cell TEC1-12706 12 V on battery and inverter terminals, where Temperature Sensors 24 and 25 of the Cerbo GX and the temperature sensor of the BMV-712 are installed, respectively.

**Figure 21:**
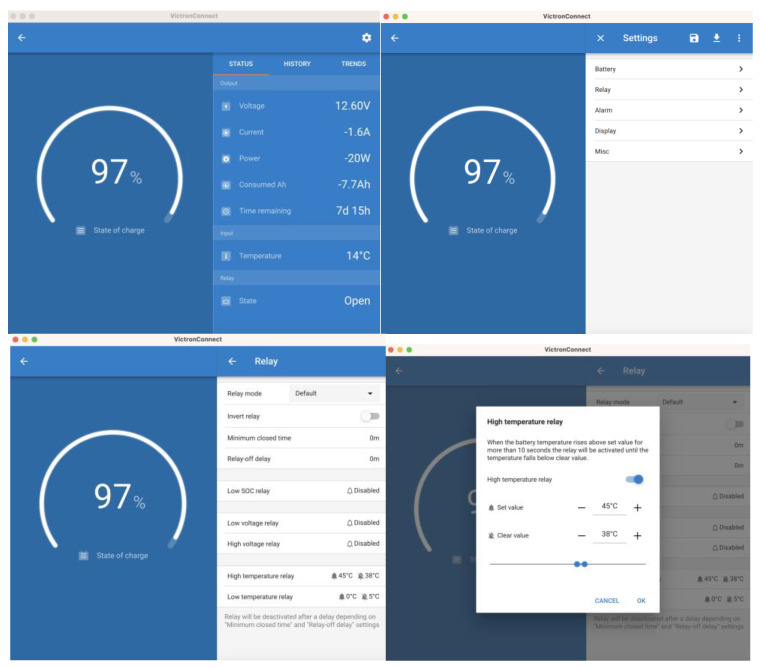
Configuration of conditions based on temperature sensor and relay of the BMS-712.

For the disconnection and isolation of the battery, especially in case of overheating and to prevent runaway phenomena, the temperature sensor ASS000100000 and a relay integrated into the BMV-712 monitor have been used to act on the two identical and synchronized KH180-type power relays. These connections and locations are shown in Figure 21. Test data are shown in Table 4.

Temperature Sensor 24 (type ASS00001000) and Relay 2 (Cerbo GX) have been used to perform the generator disconnections caused by a high-temperature alarm in the battery (Table 5).

Additionally, in the case of the inverter, if a high-temperature alarm takes place on the inverter, Temperature Sensor 25 (type ASS00001000) and Relay 2 will cause the disconnection of the generator system. Test data are shown in Table 6.

Table 7 and Table 8 show data from tests based on low-temperature alarms in the battery using Temperature Sensors 24 and 25, acting on Relay 2.

**Figure 22:**
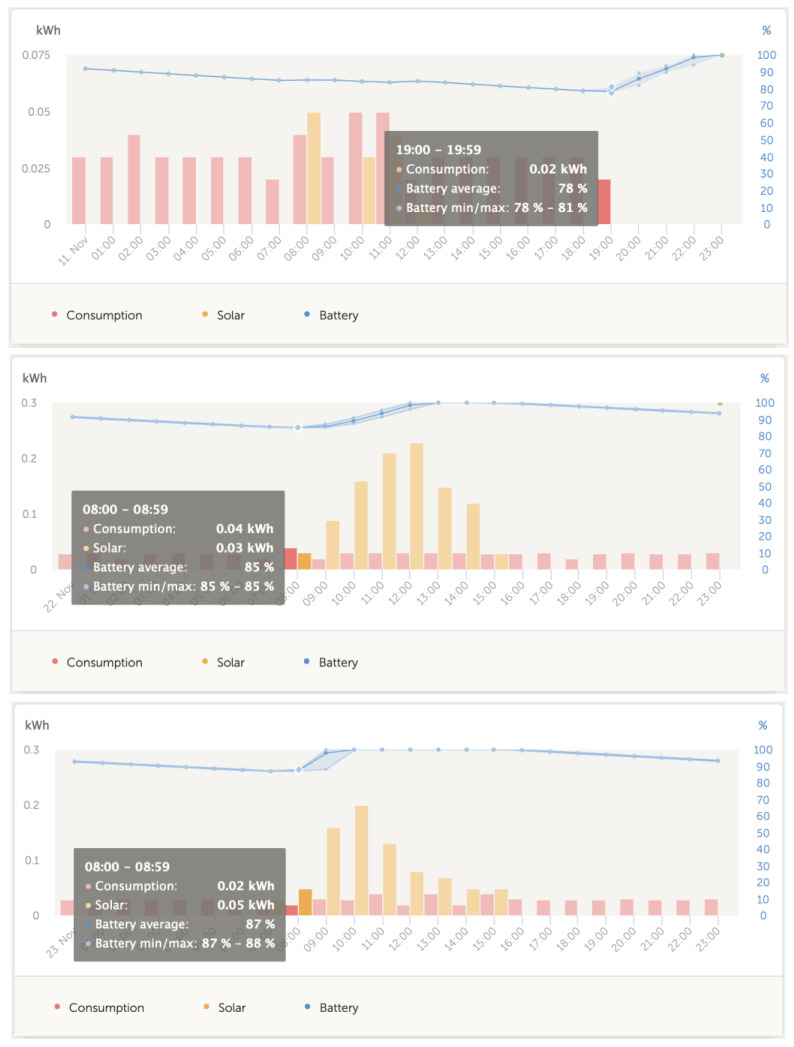
Adequate confinement SOC range for gel batteries.

Figure 22 shows that as a result of the configuration used to establish the triggering conditions of the decentralized intelligent battery management system, the operating cycle of the SOC is well within the percentage threshold values recommended for the type of batteries used in photovoltaic generation systems.

For this type of system, it is advisable to use deep-cycle batteries, but although they can withstand deep discharges occasionally, it is not advisable that this happens frequently. What is more, the manufacturers advise that the daily discharge cycles not fall below 80%.

As regards the temperature parameter, the tests carried out have also been adjusted to the safeguard threshold values of the type of gel batteries that, on the other hand, would be similar to those of lithium batteries. In [81], it is specified that the float charge voltage of gel batteries can be kept constant at temperatures up to 50 °C without any danger of thermal runaway, whereas in the case of lithium batteries, the critical value for the start of the reaction leading to thermal runaway is 60 °C.

**Figure 23:**
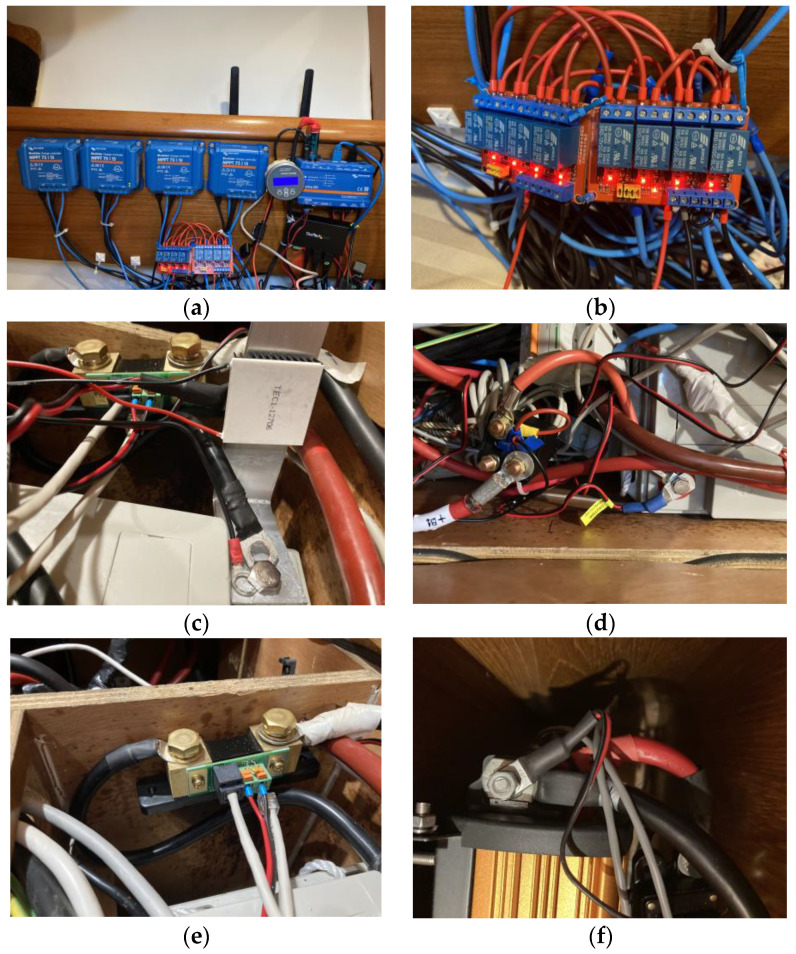
Experimental set-up. (**a**) The 4 solar chargers, the Cerbo GX, the BMV-712 monitor, and the two 4-relay modules. (**b**) The two 4-relay modules. (**c**) The Peltier cell for the induction of threshold temperature values. (**d**) The power relay controlled by the BMV-712 temperature-monitoring sensor. (**e**) The shunt of the BMV-712 device. (**f**) Temperature Sensor 25 connected to the inverter.

Figure 23 shows parts of the actual assembly where the experiments were performed.

## 4. Discussion

For real-world applications, the decentralized integrated BMS system developed in this study, from both practical hardware and software points of view, represents a significant advance.

Likewise, it can be observed that the voltage value for the start-up initiation set in the configuration window of the battery voltage function agrees with the corresponding value checked in Table 2 obtained from the downloaded data.

Likewise, in Table 3, the sporadic recharges that occur according to the conditions set in the battery current window can be checked.

The results obtained show the good performance of the decentralized BMS system with regard to its confinement to threshold values obtained in the SOC graphs in suitable conditions, preventing both an overload and a drop to levels below 80%, as can be seen in Figure 22.

The BMS used in this work presents a capacity for the adequate resolution of the thermal treatment of the batteries, as can be verified in Table 4, Table 5 and Table 6, both for the stop operations of the generation system and for the isolation of the battery under dangerous temperature conditions.

All the BMS system data were downloaded from both Amazon AWS and Influx Cloud services, which were used to store and process the VRM data.

However, there are still outstanding objectives to be solved that have to do with accurate measurements, especially the SOH, which will allow a more accurate process in terms of predictive diagnostics for the calculation of the RUL of the batteries. 

The capacity for data storage and processing in the cloud has experienced a very significant advance that could lead to the application of more complex algorithms for obtaining data with greater precision. However, in order to achieve a better analysis of the true states of batteries, battery manufacturers would need to enable easy access to certain internal battery parameters, such as internal resistance. It is known that the aging of the battery will result in the degradation of the battery capacity and an increase in the internal resistance of the battery. Therefore, the SOH of the battery can be estimated by the internal resistance as a predictor symptom. The purpose of this is to apply complex analytical techniques for SOH estimation, since with the aged capacity C_aged_ or higher internal resistance R_inc_, the SOH of the battery can be more accurately defined in order to estimate its RUL [82].

## 5. Conclusions

The designed decentralized BMS allows the increased security, supervision, monitoring, management, and optimization of any ESS system, including the ability to detect potential problems early. It provides a great capacity for handling alerts and alarms remotely, from anywhere in the world, with an immediate response on the system.

Specifically, it allows the monitoring and management of the battery state of charge, energy consumption, and energy harvesting from solar panels, generators, and grids using characteristic electrical parameters such as the voltage, current, SOC, and battery temperature. It allows the automatic start/stop of generators and disconnections remotely and in real time while providing history graphs and detailed analytical reports. This enables the predictive diagnosis of future problems and prevents ultimate system failures.

With the ever-changing real-world circumstances of off-grid installations, observations and measurements based on real-time measured parameters are crucial for optimal system performance and utilization. With VRM, intelligent rules can be easily established so that the system can cope with real problems in real time. Thanks to the information provided by the data, users of off-grid systems can adapt their energy use to better balance it with the energy captured, for example, by using the most energy-intensive devices only when there is sufficient solar irradiance.

## Figures and Tables

**Table 1:** BMV-712 relay actuation management and alarm treatment.

Actuation Management	Alarm Treatment
SoC relay (discharge floor)	Low-SoC alarm
Clear SoC relay	Clear SoC alarm
Low-voltage relay	Low-voltage alarm
Clear low-voltage relay	Clear low-voltage alarm
High-voltage relay	High-voltage alarm
Clear high-voltage relay	Clear high-voltage alarm
High-temperature relay	High-temperature alarm
Clear high-temperature relay	Clear high-temperature alarm
Low-temperature relay	Low-temperature alarm
Clear low-temperature relay	Clear low-temperature alarm

**Table 2:** Test based on voltage, current, and SOC conditions to start/stop solar generator.

Timestamp	Gateway [0]	Gateway [0]	Gateway [0]	Battery Monitor [278]	Battery Monitor [278]	Battery Monitor [278]	Battery Monitor [278]	Battery Monitor [278]
UTC (+00:00)	Relay 1 State	CCGX Relay 2 State	Generator Run Reason	Voltage	Current	Battery Temperature	State of Charge	Low Voltage Alarm
				V	A	C	%	
2023-02-17 07:17:45	Closed	Open	Battery voltage	12.18	−0.7	11	88.8	Alarm
2023-02-17 07:18:45	Closed	Open	Battery voltage	12.2	−0.2	11	88.8	Alarm
2023-02-17 07:19:45	Closed	Open	Battery voltage	12.19	−0.2	11	88.8	Alarm
2023-02-17 07:20:45	Closed	Open	Battery voltage	12.2	−0.3	11	88.8	Alarm
2023-02-17 07:21:46	Closed	Open	Battery voltage	12.2	−0.3	11	88.8	Alarm
2023-02-17 07:22:45	Closed	Open	Battery voltage	12.21	−0.2	11	88.8	Alarm
2023-02-17 07:23:45	Closed	Open	Battery voltage	12.22	−0.2	11	88.8	Alarm
2023-02-17 07:24:45	Closed	Open	Battery voltage	12.2	0.5	11	88.8	Alarm
2023-02-17 07:25:46	Closed	Open	Battery voltage	12.24	0.5	11	88.8	Alarm
2023-02-17 07:26:45	Closed	Open	Battery voltage	12.28	0.8	11	88.8	Alarm
2023-02-17 07:27:45	Closed	Open	Battery voltage	12.29	0.7	11	88.8	Alarm
2023-02-17 07:28:45	Closed	Open	Battery voltage	12.32	1	10	88.8	Alarm
2023-02-17 07:29:45	Closed	Open	Battery voltage	12.18	−3	10	88.8	Alarm
2023-02-17 07:30:46	Closed	Open	Battery voltage	12.3	1.1	10	88.8	Alarm
2023-02-17 07:31:45	Closed	Open	Battery voltage	12.34	1.2	10	88.8	Alarm
2023-02-17 07:32:45	Closed	Open	Battery voltage	12.35	1.2	10	88.8	Alarm
2023-02-17 07:33:45	Closed	Open	Battery voltage	12.37	1.5	10	88.8	Alarm
2023-02-17 07:34:45	Closed	Open	Battery voltage	12.39	1.5	10	88.8	Alarm
2023-02-17 07:35:46	Closed	Open	Battery voltage	12.35	1.5	10	88.8	Alarm
2023-02-17 07:36:45	Closed	Open	Battery voltage	12.39	1.3	11	88.8	Alarm
2023-02-17 07:36:54	Closed	Open	Battery voltage	12.4	1.8	10	88.8	No alarm
2023-02-17 07:37:45	Closed	Open	Battery voltage	12.42	1.7	10	88.8	No alarm
2023-02-17 07:38:45	Closed	Open	Battery voltage	12.45	1.9	11	88.8	No alarm
2023-02-17 07:39:46	Closed	Open	Battery voltage	12.48	1.9	10	88.8	No alarm
2023-02-17 07:40:45	Closed	Open	Battery voltage	12.34	−2.2	10	88.8	No alarm
2023-02-17 07:41:45	Closed	Open	Battery voltage	12.48	2.4	10	88.8	No alarm
2023-02-17 07:42:45	Closed	Open	Battery voltage	12.51	2.2	10	88.9	No alarm
2023-02-17 07:43:45	Closed	Open	Battery voltage	12.55	2.6	10	88.9	No alarm
2023-02-17 07:44:45	Closed	Open	Battery voltage	12.57	2.6	11	88.9	No alarm
2023-02-17 07:45:45	Closed	Open	Battery voltage	12.59	2.6	10	88.9	No alarm
2023-02-17 07:46:45	Closed	Open	Battery voltage	12.54	2.4	11	88.9	No alarm
2023-02-17 07:47:45	Closed	Open	Battery voltage	12.59	2.7	11	88.9	No alarm
2023-02-17 07:48:45	Closed	Open	Battery voltage	12.62	2.8	11	88.9	No alarm
2023-02-17 07:49:46	Closed	Open	Battery voltage	12.64	2.9	11	89	No alarm
2023-02-17 07:50:45	Closed	Open	Battery voltage	12.65	3.1	11	89	No alarm
2023-02-17 07:51:45	Closed	Open	Battery voltage	12.52	−0.9	11	89	No alarm
2023-02-17 07:52:45	Closed	Open	Battery voltage	12.64	3.3	11	89	No alarm

**Table 3:** Capture 2 in test based on voltage, current, and SOC conditions.

Timestamp	Gateway [0]	Gateway [0]	Gateway [0]	Battery Monitor [278]	Battery Monitor [278]	Battery Monitor [278]	Battery Monitor [278]	Battery Monitor [278]
UTC (+00:00)	Relay 1 State	CCGX Relay 2 State	Generator Run Reason	Voltage	Current	Battery Temperature	State of Charge	Low Voltage Alarm
				V	A	C	%	
2023-02-17 10:07:45	Closed	Open	Battery voltage	14.18	14.7	14	96.4	No alarm
2023-02-17 10:08:46	Closed	Open	Battery voltage	14.01	14.6	14	96.5	No alarm
2023-02-17 10:09:45	Closed	Open	Battery voltage	14.21	14.6	14	96.6	No alarm
2023-02-17 10:10:45	Closed	Open	Battery voltage	14.22	14.6	14	96.7	No alarm
2023-02-17 10:11:45	Closed	Open	Battery voltage	14.28	14.6	14	96.8	No alarm
2023-02-17 10:12:11	Open	Open	Stopped	14.31	14.6	14	96.8	No alarm
2023-02-17 10:12:12	Open	Open	Stopped	14.31	14.6	14	96.8	No alarm
2023-02-17 10:12:46	Open	Open	Stopped	13.03	−1.4	14	96.8	No alarm
2023-02-17 10:13:15	Closed	Open	Battery current	12.86	−5.9	14	96.8	No alarm
2023-02-17 10:13:16	Closed	Open	Battery current	12.86	−5.9	14	96.8	No alarm
2023-02-17 10:13:45	Closed	Open	Battery current	13.64	14.6	14	96.8	No alarm
2023-02-17 10:13:47	Closed	Open	Stopped	13.53	13.1	14	96.8	No alarm
2023-02-17 10:13:48	Open	Open	Stopped	13.38	11.7	14	96.8	No alarm
2023-02-17 10:14:45	Open	Open	Stopped	12.88	−1.5	14	96.8	No alarm
2023-02-17 10:15:45	Open	Open	Stopped	12.84	−1.4	14	96.8	No alarm
2023-02-17 10:16:45	Open	Open	Stopped	12.82	−1.4	14	96.8	No alarm
2023-02-17 10:17:45	Open	Open	Stopped	12.81	−1.4	14	96.8	No alarm
2023-02-17 10:18:07	Closed	Open	Battery current	12.73	−5.9	14	96.8	No alarm
2023-02-17 10:18:08	Closed	Open	Battery current	12.72	−6	14	96.8	No alarm
2023-02-17 10:18:09	Closed	Open	Battery current	12.72	−6	14	96.8	No alarm
2023-02-17 10:18:32	Open	Open	Stopped	13.55	14.5	14	96.8	No alarm
2023-02-17 10:18:33	Open	Open	Stopped	13.42	10.4	14	96.8	No alarm
2023-02-17 10:18:45	Open	Open	Stopped	12.88	−1.4	14	96.8	No alarm
2023-02-17 10:19:45	Open	Open	Stopped	12.8	−1.5	14	96.8	No alarm
2023-02-17 10:20:46	Open	Open	Stopped	12.78	−1.4	14	100	No alarm
2023-02-17 10:21:45	Open	Open	Stopped	12.77	−1.5	14	100	No alarm
2023-02-17 10:22:45	Open	Open	Stopped	12.76	−1.4	14	100	No alarm
2023-02-17 10:22:54	Closed	Open	Battery current	12.69	−5.9	14	100	No alarm
2023-02-17 10:22:55	Closed	Open	Battery current	12.68	−6	14	100	No alarm
2023-02-17 10:23:18	Closed	Open	Stopped	13.41	13.3	14	100	No alarm
2023-02-17 10:23:19	Open	Open	Stopped	13.32	12.9	14	100	No alarm
2023-02-17 10:23:45	Open	Open	Stopped	12.81	−1.4	14	100	No alarm
2023-02-17 10:24:45	Open	Open	Stopped	12.77	−1.4	14	100	No alarm
2023-02-17 10:25:45	Open	Open	Stopped	12.75	−1.4	14	100	No alarm
2023-02-17 10:26:45	Open	Open	Stopped	12.75	−1.5	14	100	No alarm
2023-02-17 10:27:43	Closed	Open	Battery current	12.66	−6	14	100	No alarm
2023-02-17 10:27:44	Closed	Open	Battery current	12.66	−6.1	14	100	No alarm
2023-02-17 10:27:45	Closed	Open	Battery current	12.66	−6.1	14	100	No alarm
2023-02-17 10:28:08	Open	Open	Stopped	13.62	14.5	14	100	No alarm
2023-02-17 10:28:09	Open	Open	Stopped	13.49	12.9	14	100	No alarm
2023-02-17 10:28:46	Open	Open	Stopped	12.78	−1.4	14	100	No alarm
2023-02-17 10:29:45	Open	Open	Stopped	12.74	−1.4	14	100	No alarm

**Table 4:** Capture in test based on high-temperature alarms using the temperature sensor ASS000100000 and relay integrated into the BMV-712 monitor.

Timestamp	Battery Monitor [278]	Battery Monitor [278]	Battery Monitor [278]	Battery Monitor [278]
UTC (+00:00)	Battery Temperature	Low Voltage Alarm	High Battery Temperature Alarm	Relay Status
	C			
2023-02-18 19:16:47	27	No alarm	No alarm	Open
2023-02-18 19:17:47	23	No alarm	No alarm	Open
2023-02-18 19:18:47	22	No alarm	No alarm	Open
2023-02-18 19:19:47	21	No alarm	No alarm	Open
2023-02-18 19:20:47	22	No alarm	No alarm	Open
2023-02-18 19:21:48	23	No alarm	No alarm	Open
2023-02-18 19:22:47	26	No alarm	No alarm	Open
2023-02-18 19:23:47	32	No alarm	No alarm	Open
2023-02-18 19:24:47	45	No alarm	Alarm	Closed
2023-02-18 19:24:58	45	No alarm	Alarm	Closed
2023-02-18 19:24:59	45	No alarm	Alarm	Closed
2023-02-18 19:25:47	44	No alarm	Alarm	Closed
2023-02-18 19:26:47	39	No alarm	Alarm	Closed
2023-02-18 19:27:12	38	No alarm	No alarm	Open
2023-02-18 19:27:47	36	No alarm	No alarm	Open
2023-02-18 19:28:48	32	No alarm	No alarm	Open
2023-02-18 19:29:47	29	No alarm	No alarm	Open
2023-02-18 19:29:53	29	No alarm	No alarm	Open
2023-02-18 19:29:56	29	No alarm	No alarm	Open
2023-02-18 19:29:58	27	No alarm	No alarm	Open
2023-02-18 19:30:47	26	No alarm	No alarm	Open
2023-02-18 19:31:47	24	No alarm	No alarm	Open
2023-02-18 19:32:47	23	No alarm	No alarm	Open
2023-02-18 19:33:47	22	No alarm	No alarm	Open
2023-02-18 19:34:47	22	No alarm	No alarm	Open
2023-02-18 19:35:47	21	No alarm	No alarm	Open
2023-02-18 19:36:47	21	No alarm	No alarm	Open
2023-02-18 19:37:47	20	No alarm	No alarm	Open
2023-02-18 19:38:47	20	No alarm	No alarm	Open
2023-02-18 19:39:47	20	No alarm	No alarm	Open
2023-02-18 19:40:47	20	No alarm	No alarm	Open
2023-02-18 19:41:47	19	No alarm	No alarm	Open
2023-02-18 19:42:47	19	No alarm	No alarm	Open
2023-02-18 19:43:47	19	No alarm	No alarm	Open
2023-02-18 19:44:47	19	No alarm	No alarm	Open
2023-02-18 19:45:47	19	No alarm	No alarm	Open
2023-02-18 19:46:47	18	No alarm	No alarm	Open

**Table 5:** Capture in test based on high-temperature alarms in the battery using Temperature Sensor 24 acting on Relay 2.

Timestamp	Gateway [0]	Gateway [0]	Temperature Sensor [24]	Solar Charger [277]	Solar Charger [278]	Solar Charger [288]	Solar Charger [289]	System Overview [0]	Temperature Sensor [25]
UTC (+00:00)	Relay 1 State	Relay 2 State	Temperature	MPPT State	MPPT State	MPPT State	MPPT State	PV - DC-Coupled	Temperature
			C					W	C
2023-02-19 10:05:48	Closed	Open	18	Voltage or current limited	Voltage or current limited	Voltage or current limited	Voltage or current limited	29	15
2023-02-19 10:06:02	Closed	Open	18	Voltage or current limited	Voltage or current limited	Voltage or current limited	Voltage or current limited	29	15
2023-02-19 10:06:49	Closed	Open	18	MPPT active	Voltage or current limited	Voltage or current limited	Voltage or current limited	135	15
2023-02-19 10:07:48	Closed	Open	18	MPPT active	Voltage or current limited	Voltage or current limited	Voltage or current limited	127	15
2023-02-19 10:08:48	Closed	Open	18	MPPT active	Voltage or current limited	Voltage or current limited	Voltage or current limited	117	15
2023-02-19 10:09:48	Closed	Open	20	MPPT active	Voltage or current limited	Voltage or current limited	Voltage or current limited	121	15
2023-02-19 10:10:48	Closed	Open	21	MPPT active	Voltage or current limited	Voltage or current limited	Voltage or current limited	113	15
2023-02-19 10:11:49	Closed	Open	20	MPPT active	Voltage or current limited	Voltage or current limited	Voltage or current limited	112	15
2023-02-19 10:12:48	Closed	Open	20	MPPT active	Voltage or current limited	Voltage or current limited	Voltage or current limited	109	15
2023-02-19 10:13:48	Closed	Open	20	MPPT active	Voltage or current limited	Voltage or current limited	Voltage or current limited	147	15
2023-02-19 10:14:48	Closed	Open	19	MPPT active	Voltage or current limited	Voltage or current limited	Voltage or current limited	108	15
2023-02-19 10:15:48	Closed	Open	19	MPPT active	Voltage or current limited	Voltage or current limited	Voltage or current limited	106	15
2023-02-19 10:16:48	Closed	Open	19	MPPT active	Voltage or current limited	Voltage or current limited	Voltage or current limited	102	15
2023-02-19 10:17:48	Closed	Open	19	MPPT active	Voltage or current limited	Voltage or current limited	Voltage or current limited	105	15
2023-02-19 10:18:48	Closed	Open	19	MPPT active	Voltage or current limited	Voltage or current limited	Voltage or current limited	109	15
2023-02-19 10:19:48	Closed	Open	19	MPPT active	Voltage or current limited	Voltage or current limited	Voltage or current limited	104	15
2023-02-19 10:20:48	Closed	Open	19	MPPT active	Voltage or current limited	Voltage or current limited	Voltage or current limited	100	15
2023-02-19 10:21:49	Closed	Open	20	MPPT active	Voltage or current limited	Voltage or current limited	Voltage or current limited	101	15
2023-02-19 10:22:48	Closed	Open	21	MPPT active	Voltage or current limited	Voltage or current limited	Voltage or current limited	150	15
2023-02-19 10:23:48	Closed	Open	23	MPPT active	Voltage or current limited	Voltage or current limited	Voltage or current limited	106	15
2023-02-19 10:24:48	Closed	Open	25	MPPT active	Voltage or current limited	Voltage or current limited	Voltage or current limited	100	15
2023-02-19 10:25:49	Closed	Open	27	MPPT active	Voltage or current limited	Voltage or current limited	Voltage or current limited	96	15
2023-02-19 10:26:48	Closed	Open	29	MPPT active	Voltage or current limited	Voltage or current limited	Voltage or current limited	96	15
2023-02-19 10:27:48	Closed	Open	30	MPPT active	Voltage or current limited	Voltage or current limited	Voltage or current limited	100	15
2023-02-19 10:28:48	Closed	Open	30	MPPT active	Voltage or current limited	Voltage or current limited	Voltage or current limited	97	15
2023-02-19 10:29:48	Closed	Open	29	MPPT active	Voltage or current limited	Voltage or current limited	Voltage or current limited	96	15
2023-02-19 10:30:48	Closed	Open	30	MPPT active	Voltage or current limited	Voltage or current limited	Voltage or current limited	93	15
2023-02-19 10:31:48	Closed	Open	39	MPPT active	Voltage or current limited	Voltage or current limited	Voltage or current limited	139	15
2023-02-19 10:31:54	Closed	Closed	40	MPPT active	Voltage or current limited	Voltage or current limited	Voltage or current limited	138	15
2023-02-19 10:32:48	Closed	Closed	44	Off	Off	Off	Off	0	15
2023-02-19 10:33:48	Closed	Closed	47	Off	Off	Off	Off	0	15
2023-02-19 10:34:49	Closed	Closed	42	Off	Off	Off	Off	0	15
2023-02-19 10:35:43	Closed	Open	38	Off	Off	Off	Off	0	15
2023-02-19 10:35:48	Closed	Open	38	Voltage or current limited	Voltage or current limited	Voltage or current limited	Voltage or current limited	61	15
2023-02-19 10:36:46	Closed	Open	35	Voltage or current limited	Voltage or current limited	Voltage or current limited	Voltage or current limited	139	15
2023-02-19 10:36:48	Closed	Open	35	Voltage or current limited	Voltage or current limited	Voltage or current limited	Voltage or current limited	138	15
2023-02-19 10:37:48	Closed	Open	32	Voltage or current limited	Voltage or current limited	Voltage or current limited	Voltage or current limited	131	15
2023-02-19 10:38:49	Closed	Open	30	Voltage or current limited	Voltage or current limited	Voltage or current limited	Voltage or current limited	128	20
2023-02-19 10:39:48	Closed	Open	28	Voltage or current limited	Voltage or current limited	Voltage or current limited	Voltage or current limited	128	25
2023-02-19 10:40:48	Closed	Open	25	Voltage or current limited	Voltage or current limited	Voltage or current limited	Voltage or current limited	186	25
2023-02-19 10:41:48	Closed	Open	24	Voltage or current limited	Voltage or current limited	Voltage or current limited	Voltage or current limited	126	25
2023-02-19 10:42:48	Closed	Open	22	Voltage or current limited	Voltage or current limited	Voltage or current limited	Voltage or current limited	125	24
2023-02-19 10:43:48	Closed	Open	21	Voltage or current limited	Voltage or current limited	Voltage or current limited	Voltage or current limited	123	23
2023-02-19 10:44:49	Closed	Open	20	Voltage or current limited	Voltage or current limited	Voltage or current limited	Voltage or current limited	122	21
2023-02-19 10:45:48	Closed	Open	19	Voltage or current limited	Voltage or current limited	Voltage or current limited	Voltage or current limited	122	19
2023-02-19 10:46:48	Closed	Open	19	Voltage or current limited	Voltage or current limited	Voltage or current limited	Voltage or current limited	119	19
2023-02-19 10:47:48	Closed	Open	18	Voltage or current limited	Voltage or current limited	Voltage or current limited	Voltage or current limited	118	19
2023-02-19 10:48:48	Closed	Open	18	Voltage or current limited	Voltage or current limited	Voltage or current limited	Voltage or current limited	118	19
2023-02-19 10:49:48	Closed	Open	18	Voltage or current limited	Voltage or current limited	Voltage or current limited	Voltage or current limited	112	19
2023-02-19 10:50:49	Closed	Open	18	Voltage or current limited	Voltage or current limited	Voltage or current limited	Voltage or current limited	112	19

**Table 6:** Capture in test based on high-temperature alarms in the inverter using Temperature Sensor 25, acting on Relay 2.

Timestamp	Gateway [0]	Gateway [0]	Temperature Sensor [25]	Solar Charger [277]	Solar Charger [278]	Solar Charger [288]	Solar Charger [289]	System Overview [0]	Temperature Sensor [24]
UTC (+00:00)	Relay 1 State	Relay 2 State	Temperature	MPPT State	MPPT State	MPPT State	MPPT State	PV - DC-Coupled	Temperature
			C					W	C
2023-02-19 10:53:48	Closed	Open	21	Voltage or current limited	Voltage or current limited	Voltage or current limited	Voltage or current limited	168	17
2023-02-19 10:54:48	Closed	Open	22	Voltage or current limited	Voltage or current limited	Voltage or current limited	Voltage or current limited	110	17
2023-02-19 10:55:49	Closed	Open	23	Voltage or current limited	Voltage or current limited	Voltage or current limited	Voltage or current limited	112	17
2023-02-19 10:56:48	Closed	Open	24	Voltage or current limited	Voltage or current limited	Voltage or current limited	Voltage or current limited	109	17
2023-02-19 10:57:48	Closed	Open	25	Voltage or current limited	Voltage or current limited	Voltage or current limited	Voltage or current limited	106	16
2023-02-19 10:58:48	Closed	Open	27	Voltage or current limited	Voltage or current limited	Voltage or current limited	Voltage or current limited	106	16
2023-02-19 10:59:48	Closed	Open	29	Voltage or current limited	Voltage or current limited	Voltage or current limited	Voltage or current limited	106	16
2023-02-19 11:00:49	Closed	Open	31	Voltage or current limited	Voltage or current limited	Voltage or current limited	Voltage or current limited	105	16
2023-02-19 11:01:48	Closed	Open	32	Voltage or current limited	Voltage or current limited	Voltage or current limited	Voltage or current limited	103	16
2023-02-19 11:02:39	Closed	Open	33	Voltage or current limited	Voltage or current limited	Voltage or current limited	Voltage or current limited	154	16
2023-02-19 11:02:48	Closed	Open	33	Voltage or current limited	Voltage or current limited	MPPT active	MPPT active	119	16
2023-02-19 11:03:03	Closed	Open	34	Voltage or current limited	Voltage or current limited	Voltage or current limited	Voltage or current limited	105	16
2023-02-19 11:03:10	Closed	Open	34	Voltage or current limited	Voltage or current limited	Voltage or current limited	Voltage or current limited	88	16
2023-02-19 11:03:48	Closed	Open	34	Voltage or current limited	Voltage or current limited	MPPT active	MPPT active	76	16
2023-02-19 11:04:48	Closed	Open	35	Voltage or current limited	Voltage or current limited	MPPT active	MPPT active	81	16
2023-02-19 11:05:17	Closed	Open	36	Voltage or current limited	Voltage or current limited	Voltage or current limited	Voltage or current limited	103	16
2023-02-19 11:05:48	Closed	Open	36	Voltage or current limited	Voltage or current limited	Voltage or current limited	Voltage or current limited	100	16
2023-02-19 11:06:48	Closed	Open	37	Voltage or current limited	Voltage or current limited	Voltage or current limited	Voltage or current limited	157	16
2023-02-19 11:07:48	Closed	Open	37	Voltage or current limited	Voltage or current limited	Voltage or current limited	Voltage or current limited	99	16
2023-02-19 11:08:45	Closed	Open	37	Voltage or current limited	Voltage or current limited	Voltage or current limited	Voltage or current limited	84	16
2023-02-19 11:08:48	Closed	Open	37	Voltage or current limited	Voltage or current limited	MPPT active	MPPT active	79	16
2023-02-19 11:09:49	Closed	Open	38	Voltage or current limited	Voltage or current limited	Voltage or current limited	Voltage or current limited	99	16
2023-02-19 11:09:52	Closed	Open	38	Voltage or current limited	Voltage or current limited	Voltage or current limited	Voltage or current limited	99	16
2023-02-19 11:10:31	Closed	Open	38	Voltage or current limited	Voltage or current limited	Voltage or current limited	Voltage or current limited	87	16
2023-02-19 11:10:48	Closed	Open	38	Voltage or current limited	Voltage or current limited	MPPT active	MPPT active	67	16
2023-02-19 11:11:45	Closed	Open	39	Voltage or current limited	Voltage or current limited	Voltage or current limited	Voltage or current limited	96	16
2023-02-19 11:11:48	Closed	Open	39	Voltage or current limited	Voltage or current limited	Voltage or current limited	Voltage or current limited	97	16
2023-02-19 11:11:50	Closed	Closed	40	Voltage or current limited	Voltage or current limited	Voltage or current limited	Voltage or current limited	96	16
2023-02-19 11:11:52	Closed	Closed	40	Off	Off	Voltage or current limited	Voltage or current limited	0	16
2023-02-19 11:12:48	Closed	Closed	41	Off	Off	Off	Off	0	16
2023-02-19 11:13:48	Closed	Closed	41	Off	Off	Off	Off	0	16
2023-02-19 11:14:48	Closed	Closed	42	Off	Off	Off	Off	0	16
2023-02-19 11:15:48	Closed	Closed	42	Off	Off	Off	Off	0	16
2023-02-19 11:16:48	Closed	Closed	39	Off	Off	Off	Off	0	16
2023-02-19 11:16:56	Closed	Open	38	Off	Off	Off	Off	0	16
2023-02-19 11:17:41	Closed	Open	36	Voltage or current limited	Voltage or current limited	Voltage or current limited	Voltage or current limited	116	16
2023-02-19 11:17:48	Closed	Open	35	Voltage or current limited	Voltage or current limited	Voltage or current limited	Voltage or current limited	110	16
2023-02-19 11:18:48	Closed	Open	33	Voltage or current limited	Voltage or current limited	Voltage or current limited	Voltage or current limited	100	16
2023-02-19 11:19:48	Closed	Open	31	Voltage or current limited	Voltage or current limited	Voltage or current limited	Voltage or current limited	156	16
2023-02-19 11:20:48	Closed	Open	29	Voltage or current limited	Voltage or current limited	Voltage or current limited	Voltage or current limited	91	16
2023-02-19 11:21:25	Closed	Open	28	Voltage or current limited	Voltage or current limited	Voltage or current limited	Voltage or current limited	51	16
2023-02-19 11:21:34	Closed	Open	28	Voltage or current limited	Voltage or current limited	Voltage or current limited	Voltage or current limited	29	16
2023-02-19 11:21:48	Closed	Open	27	Voltage or current limited	Voltage or current limited	Voltage or current limited	Voltage or current limited	28	16
2023-02-19 11:22:23	Closed	Open	28	Voltage or current limited	Voltage or current limited	Voltage or current limited	Voltage or current limited	29	16
2023-02-19 11:22:33	Closed	Open	29	Voltage or current limited	Voltage or current limited	Voltage or current limited	Voltage or current limited	31	16
2023-02-19 11:22:48	Closed	Open	28	Voltage or current limited	Voltage or current limited	Voltage or current limited	Voltage or current limited	30	16
2023-02-19 11:23:34	Closed	Open	26	Voltage or current limited	Voltage or current limited	Voltage or current limited	Voltage or current limited	30	21
2023-02-19 11:23:43	Closed	Open	26	Voltage or current limited	Voltage or current limited	Voltage or current limited	Voltage or current limited	30	22
2023-02-19 11:23:45	Closed	Open	26	Voltage or current limited	Voltage or current limited	Voltage or current limited	Voltage or current limited	70	23
2023-02-19 11:23:48	Closed	Open	26	Voltage or current limited	Voltage or current limited	Voltage or current limited	MPPT active	86	23
2023-02-19 11:24:48	Closed	Open	24	Voltage or current limited	Voltage or current limited	Voltage or current limited	Voltage or current limited	81	25
2023-02-19 11:25:48	Closed	Open	22	Voltage or current limited	Voltage or current limited	Voltage or current limited	MPPT active	79	24
2023-02-19 11:26:48	Closed	Open	21	Voltage or current limited	Voltage or current limited	Voltage or current limited	MPPT active	78	22
2023-02-19 11:27:49	Closed	Open	20	Voltage or current limited	Voltage or current limited	Voltage or current limited	MPPT active	80	21
2023-02-19 11:28:48	Closed	Open	19	Voltage or current limited	Voltage or current limited	Voltage or current limited	MPPT active	81	20
2023-02-19 11:29:48	Closed	Open	19	Voltage or current limited	Voltage or current limited	Voltage or current limited	MPPT active	80	20
2023-02-19 11:30:48	Closed	Open	18	Voltage or current limited	Voltage or current limited	Voltage or current limited	MPPT active	80	19
2023-02-19 11:31:48	Closed	Open	18	Voltage or current limited	Voltage or current limited	Voltage or current limited	MPPT active	82	19
2023-02-19 11:32:48	Closed	Open	18	Voltage or current limited	Voltage or current limited	Voltage or current limited	MPPT active	121	19
2023-02-19 11:33:48	Closed	Open	17	Voltage or current limited	Voltage or current limited	Voltage or current limited	MPPT active	75	19
2023-02-19 11:34:48	Closed	Open	17	Voltage or current limited	Voltage or current limited	Voltage or current limited	MPPT active	68	18
2023-02-19 11:35:48	Closed	Open	17	Voltage or current limited	Voltage or current limited	Voltage or current limited	MPPT active	78	18
2023-02-19 11:36:49	Closed	Open	17	Voltage or current limited	Voltage or current limited	Voltage or current limited	MPPT active	77	18
2023-02-19 11:37:48	Closed	Open	17	Voltage or current limited	Voltage or current limited	Voltage or current limited	MPPT active	71	18

**Table 7:** Capture in test based on low-temperature alarms in the battery using Temperature Sensor 24, acting on Relay 2.

Timestamp	Gateway [0]	Gateway [0]	Temperature Sensor [24]	Solar Charger [277]	Solar Charger [278]	Solar Charger [288]	Solar Charger [289]	Temperature Sensor [25]
UTC (+00:00)	Relay 1 State	CCGX Relay 2 State	Temperature	MPPT State	MPPT State	MPPT State	MPPT State	Temperature
			C					C
2023-02-24 08:16:56	Closed	Open	13	MPPT active	MPPT active	MPPT active	MPPT active	12
2023-02-24 08:17:55	Closed	Open	13	MPPT active	MPPT active	MPPT active	MPPT active	12
2023-02-24 08:18:55	Closed	Open	9	MPPT active	MPPT active	MPPT active	MPPT active	13
2023-02-24 08:19:55	Closed	Open	6	MPPT active	MPPT active	MPPT active	MPPT active	13
2023-02-24 08:20:55	Closed	Open	4	MPPT active	MPPT active	MPPT active	MPPT active	13
2023-02-24 08:21:55	Closed	Open	3	MPPT active	MPPT active	MPPT active	MPPT active	13
2023-02-24 08:22:56	Closed	Open	2	MPPT active	MPPT active	MPPT active	MPPT active	13
2023-02-24 08:23:55	Closed	Open	2	MPPT active	MPPT active	MPPT active	MPPT active	13
2023-02-24 08:24:55	Closed	Open	3	MPPT active	MPPT active	MPPT active	MPPT active	13
2023-02-24 08:25:55	Closed	Open	2	MPPT active	MPPT active	MPPT active	MPPT active	13
2023-02-24 08:26:56	Closed	Open	1	MPPT active	MPPT active	MPPT active	MPPT active	13
2023-02-24 08:27:31	Closed	Closed	0	MPPT active	MPPT active	MPPT active	MPPT active	13
2023-02-24 08:27:55	Closed	Closed	0	Off	Off	Off	Off	13
2023-02-24 08:28:55	Closed	Closed	0	Off	Off	Off	Off	13
2023-02-24 08:29:55	Closed	Closed	0	Off	Off	Off	Off	13
2023-02-24 08:30:55	Closed	Closed	0	Off	Off	Off	Off	13
2023-02-24 08:31:56	Closed	Closed	0	Off	Off	Off	Off	13
2023-02-24 08:32:37	Closed	Open	2	Off	Off	Off	Off	13
2023-02-24 08:32:55	Closed	Open	2	MPPT active	MPPT active	MPPT active	MPPT active	13
2023-02-24 08:33:55	Closed	Open	4	MPPT active	MPPT active	MPPT active	MPPT active	13
2023-02-24 08:34:55	Closed	Open	5	MPPT active	MPPT active	MPPT active	MPPT active	13
2023-02-24 08:35:55	Closed	Open	6	MPPT active	MPPT active	MPPT active	MPPT active	13
2023-02-24 08:36:55	Closed	Open	7	MPPT active	MPPT active	MPPT active	MPPT active	13
2023-02-24 08:37:56	Closed	Open	8	MPPT active	MPPT active	MPPT active	MPPT active	13
2023-02-24 08:38:55	Closed	Open	8	MPPT active	MPPT active	MPPT active	MPPT active	13
2023-02-24 08:39:55	Closed	Open	9	MPPT active	MPPT active	MPPT active	MPPT active	13
2023-02-24 08:40:55	Closed	Open	9	MPPT active	MPPT active	MPPT active	MPPT active	13
2023-02-24 08:41:55	Closed	Open	9	MPPT active	MPPT active	MPPT active	MPPT active	13
2023-02-24 08:42:55	Closed	Open	10	MPPT active	MPPT active	MPPT active	MPPT active	13
2023-02-24 08:43:55	Closed	Open	10	MPPT active	MPPT active	MPPT active	MPPT active	13
2023-02-24 08:44:55	Closed	Open	10	MPPT active	MPPT active	MPPT active	MPPT active	13
2023-02-24 08:45:55	Closed	Open	10	MPPT active	MPPT active	MPPT active	MPPT active	13
2023-02-24 08:46:55	Closed	Open	10	MPPT active	MPPT active	MPPT active	MPPT active	13
2023-02-24 08:47:55	Closed	Open	10	MPPT active	MPPT active	MPPT active	MPPT active	13
2023-02-24 08:48:55	Closed	Open	11	MPPT active	MPPT active	MPPT active	MPPT active	13
2023-02-24 08:49:56	Closed	Open	11	MPPT active	MPPT active	MPPT active	MPPT active	13
2023-02-24 08:50:55	Closed	Open	11	MPPT active	MPPT active	MPPT active	MPPT active	13
2023-02-24 08:51:55	Closed	Open	11	MPPT active	MPPT active	MPPT active	MPPT active	13
2023-02-24 08:52:55	Closed	Open	11	MPPT active	MPPT active	MPPT active	MPPT active	13
2023-02-24 08:53:55	Closed	Open	11	MPPT active	MPPT active	MPPT active	MPPT active	13
2023-02-24 08:54:55	Closed	Open	11	MPPT active	MPPT active	MPPT active	MPPT active	14
2023-02-24 08:55:55	Closed	Open	11	MPPT active	MPPT active	MPPT active	MPPT active	14
2023-02-24 08:56:55	Closed	Open	11	MPPT active	MPPT active	MPPT active	MPPT active	14
2023-02-24 08:57:47	Closed	Open	12	MPPT active	MPPT active	MPPT active	MPPT active	14

**Table 8:** Capture in test based on low-temperature alarms in the inverter using Temperature Sensor 25, acting on Relay 2.

Timestamp	Gateway [0]	Gateway [0]	Temperature Sensor [25]	Solar Charger [277]	Solar Charger [278]	Solar Charger [288]	Solar Charger [289]	Temperature Sensor [24]
UTC (+00:00)	Relay 1 State	Relay 2 State	Temperature	MPPT State	MPPT State	MPPT State	MPPT State	Temperature
			C					C
2023-02-24 07:26:56	Closed	Open	13	MPPT active	MPPT active	MPPT active	MPPT active	13
2023-02-24 07:27:55	Closed	Open	13	MPPT active	MPPT active	MPPT active	MPPT active	13
2023-02-24 07:28:55	Closed	Open	13	MPPT active	MPPT active	MPPT active	MPPT active	13
2023-02-24 07:29:55	Closed	Open	13	MPPT active	MPPT active	MPPT active	MPPT active	13
2023-02-24 07:30:55	Closed	Open	13	MPPT active	Voltage or current limited	MPPT active	MPPT active	13
2023-02-24 07:31:56	Closed	Open	11	MPPT active	Off	MPPT active	MPPT active	13
2023-02-24 07:32:55	Closed	Open	9	MPPT active	MPPT active	MPPT active	MPPT active	13
2023-02-24 07:33:55	Closed	Open	7	MPPT active	MPPT active	MPPT active	MPPT active	13
2023-02-24 07:34:55	Closed	Open	5	MPPT active	MPPT active	MPPT active	MPPT active	13
2023-02-24 07:35:55	Closed	Open	4	MPPT active	MPPT active	MPPT active	MPPT active	13
2023-02-24 07:36:55	Closed	Open	3	MPPT active	MPPT active	MPPT active	MPPT active	13
2023-02-24 07:37:56	Closed	Open	3	MPPT active	MPPT active	MPPT active	MPPT active	13
2023-02-24 07:38:55	Closed	Open	2	MPPT active	MPPT active	MPPT active	MPPT active	13
2023-02-24 07:39:55	Closed	Open	2	MPPT active	Voltage or current limited	MPPT active	MPPT active	13
2023-02-24 07:40:55	Closed	Open	2	MPPT active	Off	MPPT active	MPPT active	13
2023-02-24 07:41:55	Closed	Open	2	MPPT active	MPPT active	MPPT active	MPPT active	13
2023-02-24 07:42:55	Closed	Open	3	MPPT active	MPPT active	MPPT active	MPPT active	13
2023-02-24 07:43:56	Closed	Open	2	MPPT active	MPPT active	MPPT active	MPPT active	13
2023-02-24 07:44:41	Closed	Closed	0	MPPT active	MPPT active	MPPT active	MPPT active	13
2023-02-24 07:44:55	Closed	Closed	0	Off	Off	Off	Off	13
2023-02-24 07:45:55	Closed	Closed	0	Off	Off	Off	Off	13
2023-02-24 07:46:55	Closed	Closed	−1	Off	Off	Off	Off	13
2023-02-24 07:47:55	Closed	Closed	−1	Off	Off	Off	Off	13
2023-02-24 07:48:55	Closed	Closed	−1	Off	Off	Off	Off	13
2023-02-24 07:49:55	Closed	Closed	0	Off	Off	Off	Off	13
2023-02-24 07:50:55	Closed	Closed	0	Off	Off	Off	Off	13
2023-02-24 07:51:55	Closed	Closed	0	Off	Off	Off	Off	13
2023-02-24 07:52:55	Closed	Closed	0	Off	Off	Off	Off	13
2023-02-24 07:53:55	Closed	Closed	0	Off	Off	Off	Off	13
2023-02-24 07:54:55	Closed	Closed	1	Off	Off	Off	Off	13
2023-02-24 07:55:56	Closed	Closed	1	Off	Off	Off	Off	13
2023-02-24 07:56:18	Closed	Open	2	Off	Off	Off	Off	13
2023-02-24 07:56:55	Closed	Open	2	MPPT active	MPPT active	MPPT active	MPPT active	13
2023-02-24 07:57:55	Closed	Open	2	MPPT active	MPPT active	MPPT active	MPPT active	13
2023-02-24 07:58:55	Closed	Open	3	MPPT active	MPPT active	MPPT active	MPPT active	13
2023-02-24 07:59:55	Closed	Open	3	MPPT active	MPPT active	MPPT active	MPPT active	13
2023-02-24 08:00:56	Closed	Open	4	MPPT active	MPPT active	MPPT active	MPPT active	13
2023-02-24 08:01:55	Closed	Open	4	MPPT active	MPPT active	MPPT active	MPPT active	13
2023-02-24 08:02:55	Closed	Open	4	MPPT active	MPPT active	MPPT active	MPPT active	13
2023-02-24 08:03:55	Closed	Open	5	MPPT active	MPPT active	MPPT active	MPPT active	13
2023-02-24 08:04:55	Closed	Open	7	MPPT active	MPPT active	MPPT active	MPPT active	13
2023-02-24 08:05:55	Closed	Open	7	MPPT active	MPPT active	MPPT active	MPPT active	13
2023-02-24 08:06:56	Closed	Open	8	MPPT active	MPPT active	MPPT active	MPPT active	13
2023-02-24 08:07:55	Closed	Open	9	MPPT active	MPPT active	MPPT active	MPPT active	13

## Data Availability

The data presented in this study are available on request from the corresponding author.

## Supplementary Materials

The following supporting information can be downloaded at https://www.mdpi.com/article/10.3390/s23073417/s1, Tables and Figures regarding the BMS operation procedures and configuration parameters used in this study.

## Abbreviations

AGM	Absorbent Glass Mat
BMS	Battery management system
BMV	Battery Monitoring Victron
Cap	Battery’s current capacity
C-rate	Discharge velocity
COM	Common pin
DC+	DC power supply positive pole
DC−	DC power supply negative pole
DOD	Depth of discharge
DVCC	Distributed voltage and current control
ESS	Energy storage system
I	Current parameter
KOH	Potassium hydroxide
LiFePO4	Lithium iron phosphate
MPPT	Maximum power point tracking controller
NC	Normally closed pin
NiCd	Nickel–cadmium
NO	Normally opened pin
OCV	Open-circuit voltage
PNs	Petri Nets
PRV	Pressure relief valve
PV	Photovoltaic
R	Resistance
RC	Resistance capacitor
RUL	Battery remaining useful life
SCC	Solar charge controller
SCS	Shared current sensor
SOC	State of charge
SOE	State of energy
SOH	State of health
SOP	State of power
STS	Shared temperature sensor
S1	Switch Channel 1 Relay
S2	Switch Channel 2 Relay
T	Temperature parameter
V	Voltage parameter
VRLA	Valve-Regulated Lead–Acid
VRM	Victron Remote Monitoring
