# Supplementary material for: Distributed Intelligent Battery Management System Using a Real-World Cloud Computing System
Source: Sensors (Basel). 2023 Mar 24;23(7):3417. doi: 10.3390/s23073417 (PMC10098843; doi:10.3390/s23073417)
Supplement: Supplementary file 1 [file sensors-23-03417-s001.zip › sensors-2276244-supplementary.pdf]

This supplementary file shows the BMS operation procedures and configuration parameters used in this study.

Supplementary S1: Actuation of the Cerbo GX Relays by Temperature Parameters

Relays 1 and 2 integrated in the Cerbo GX can be configured to be activated and deactivated according to the temperature threshold values that are reached, both for high and low temperatures (Figure S1).

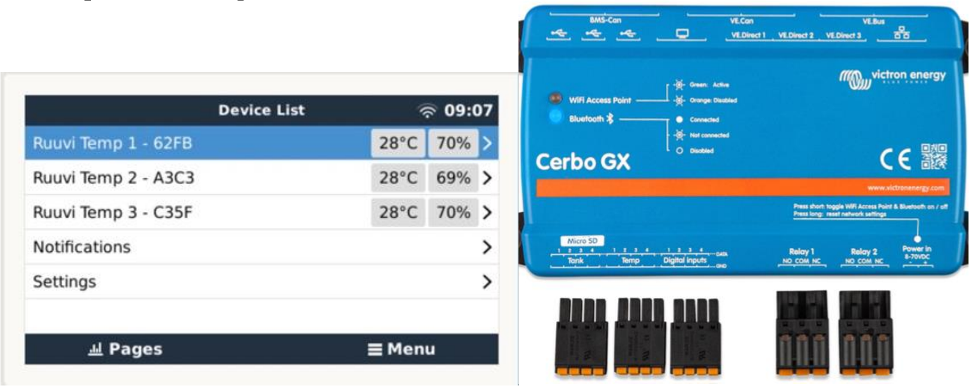

Figure S1. Ruuvi temperature sensor configuration window.

The temperature relay is controlled from: Settings menu -> Relay -> Function (Relay 1/2) -> Temperature. When enabled, the temperature control rules menu will show up in the relay menu and within any detected temperature sensors (Figure S2).

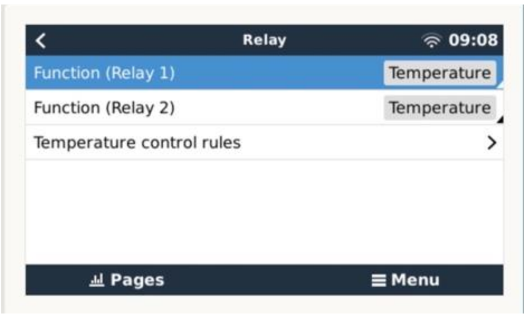

Figure S2. Relay 1 and Relay 2 configuration window.

The relay can be managed by each temperature sensor. The relay can be controlled by the temperature sensor of your choice (Figure S3). Inactive temperature sensors will show the phrase "No actions" on their display. Every temperature sensor's relay control can be enabled or disabled from this menu. With two relays available on the Cerbo GX controller, it is possible to control both relays with just one temperature sensor. Additionally, it is feasible to have a single relay controlled by a variety of temperature sensors.

As an illustration, a Cerbo GX that only uses both of its lithium battery heaters simultaneously when necessary.

1. In the Relay -> Temperature control rules -> Temperature sensor menu.
2. Enable the relay activation by temperature.
3. Assign the relay control to Relay 1.
4. Set the activation value to 5 degrees
5. Set the deactivation value to 10 degrees.

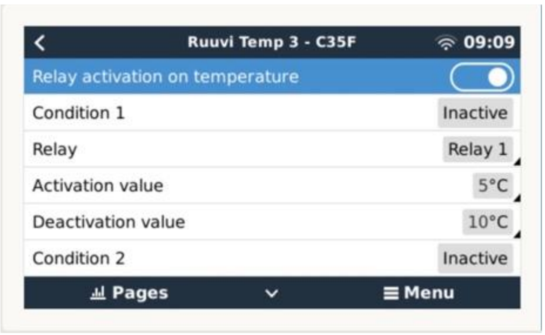

Figure S3. Example configuration window.

If this is not enough to keep the battery temperature above 5 degrees, you may also want to connect a second heater contactor to Relay 2 (Figure S4).

1. Scroll down in the menu to Condition 2.
2. Assign the relay control to Relay 2.
3. Set the activation value to 4 degrees
4. Set the deactivation value to 6 degrees.

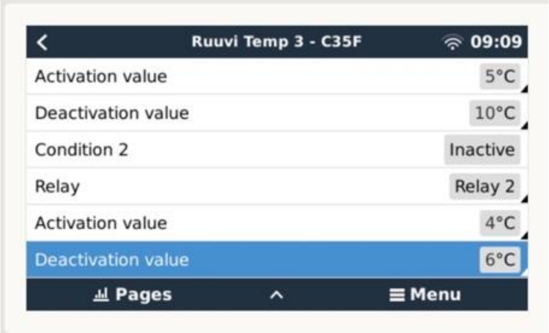

Figure S4. Example extension configuration window.

This means that Relay 1 will turn on if the battery temperature falls below 5 degrees. Relay 2 will turn on the second heater if the temperature stays below 5 to 4 degrees. If this is insufficient to raise the temperature to 6 degrees, Relay 2 will be turned off and Relay 1 will stay on until the battery temperature reaches 10 degrees. Physical switching contacts are available for both relays in Normally Open and Normally Closed configurations. An additional contactor may optionally be required in the event that the power requirements to actuate the heaters exceed the relay power limit specification.

Supplementary S2: Automatic start/stop of the general generator systems

The Cerbo GX internal relays can be used to automatically start/stop any generator system. These relays can be "triggered" with a set of user-defined conditions. Of the two relays available, Relay 1 can be used directly to perform the automatic start/stop function of the generator. The wiring between Cerbo GX must be "permanent" to keep the generator running; and also, to allow automatic "stop" when the automatic stop parameters are met.

To do this, select: Settings → Relay → "Generator start/stop" and configure the latter function. The sequence of activation of configuration windows is shown in Figures A5, A6 and A7.

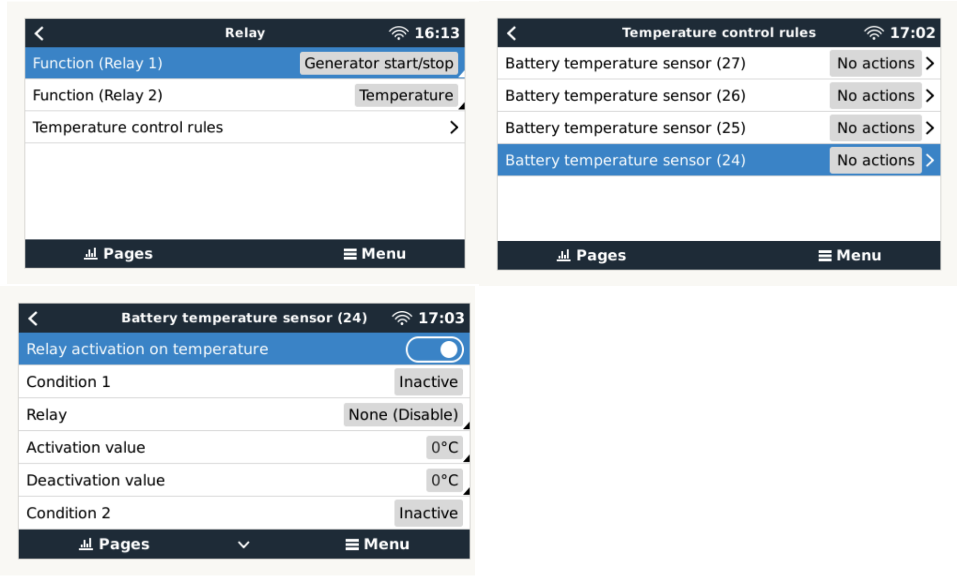

Figure S5. Sequence of activation of configuration windows.

Once activated, under: Settings→ Generator start/stop next you can find all settings related to the Generator auto start/stop function in the main menu.

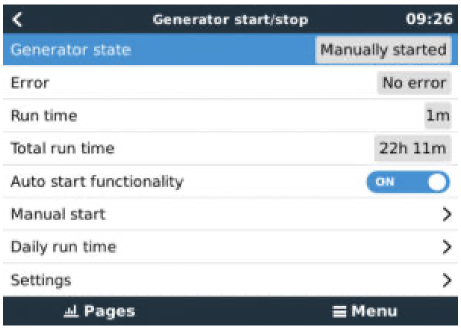

Figure S6. Generator start/stop configuration window

Where additionally different parameters can be monitored:

- Generator status: Current status of the generator.
- Error: Description of the error.
- Running time: Generator running time since last start.
- Total running time: Accumulated running time since first start.
- Time to next test run: When the "test run" option is enabled, this item shows the time remaining until the next test run.
- Daily run time: Runtime history for the last 30 days.
- Settings: This is the access to all other options.

Conditions Parameters that can be defined by the user to activate the automatic start of the generator system.

To properly configure the system from the main menu the following sequence of operations must be followed a: Settings→ Relay → Generator start and stop→ Settings→ Settings→ Conditions.

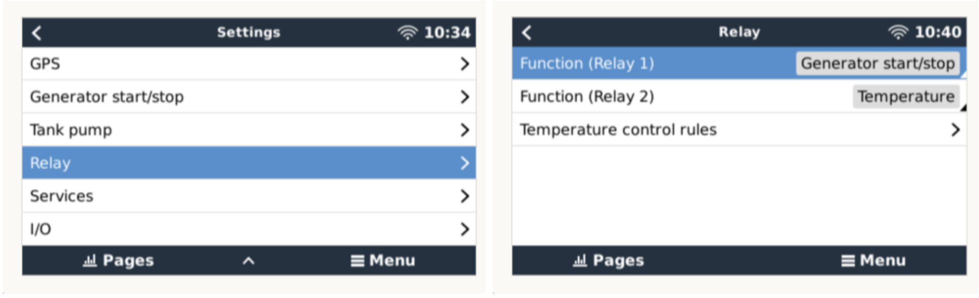

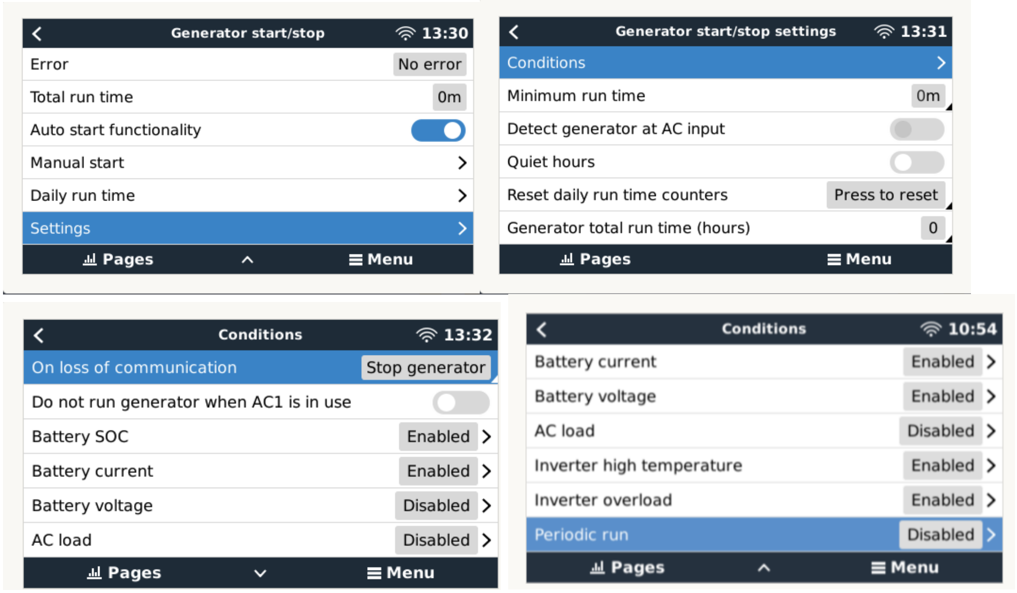

Figure S7. Sequence of activation of Generator start/stop configuration windows.

The explanations that follow refer to the items contained in Figures A7:

- If communication is lost: If communication between the Cerbo GX/Cerbo GX and the selected battery monitor, and/or the inverter/charger VE.bus is ever interrupted and parameters have been set that depend on this information - choose from the following actions:

- Stop generator: if the generator is running, it will stop. (default)

- Start generator: If the generator is not running, it will start.

- Keep running: If the generator is running at the time of data loss, this setting will keep it running.

In the same Conditions settings window (Figure S8), the user can define the following parameters to specifically trigger the automatic start/stop of the generator:

- State of Charge (SoC).
- Battery current
- Battery voltage
- AC load
- Inverter high temperature
- Inverter overload
- Periodic test start-up

(* The measured value here will be the total AC consumption of the system).

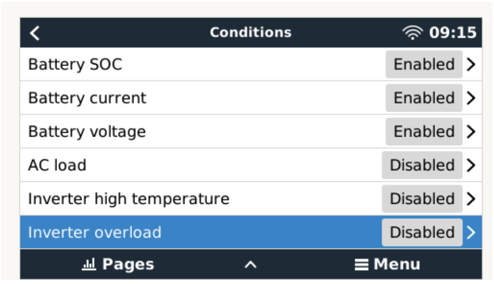

Figure S8. Inverter high temperature/ overload configuration window.

Priority is given to the condition parameters in the order shown above in Figure S8. Only the condition with the highest priority will be displayed as active when multiple conditions are satisfied simultaneously. The generator will still be evaluated even if it is already running under all enabled conditions. An unmet parameter of a lower priority condition will keep the generator running after the active condition has been satisfied.

If for example the Battery SOC condition is selected, we can enable the start/stop of the Generator by setting the conditions by specific determined values.

Relays activated by the battery state of charge (Figure S9):
- Use the battery state of charge value to start/stop: This option can be set to on or off.
- Start when battery state of charge is less than: Set the depletion level for automatic start.
- Start value during Quiet Periods: During Quiet Periods it may be appropriate to delay the automatic start until it is unavoidable. If a Quiet Period has been defined set a lower automatic start level, to a more critical level.
- Stop when the battery state of charge is higher than: Set the recharge level for automatic stop.
- Stop value during silent periods: If a Silent period has been defined, set a lower automatic stop level so that the generator running time is shorter.

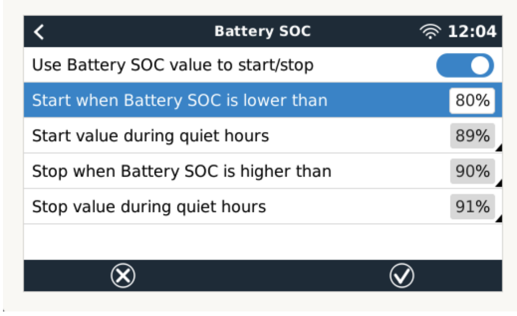

Figure S9. SOC configuration window.

Battery current activated relays (Figure S10):
- Use the battery current value to start/stop: This option can be set to on or off.
- Start when battery current is greater than: When experiencing a high AC demand that will soon drain the battery, the generator can automatically start to help meet that demand. Set the current level at which this will happen.
- Start value during Quiet Periods: If a Quiet Period has been defined set a higher current value, at a more critical level.
- Start when condition is met for: Set an interval prior to automatic start. Some AC devices will draw a high but short peak current at startup. Forcing a time interval prior to automatic start will prevent the generator from starting unnecessarily.
- Stop when the battery current is below: Set the current for automatic stop.
- Stop value during quiet periods: If a Quiet period has been defined, set a higher current level so that the running time of the generator is shorter.
- Stop when condition is met for: Set a time interval to ensure that the current reduction condition is stable.

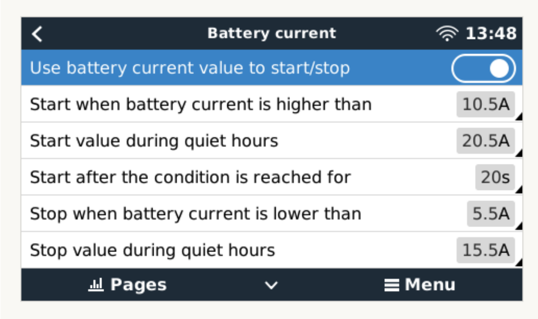

Figure S10. Battery current configuration window.

Relays activated by battery voltage (Figure S11):
- Use the battery voltage value to start/stop: This option can be set to on or off.
- Start when the battery voltage is below: When the battery voltage drops below the entered value the relay will start the generator.
- Start value during silent periods: If a Silent period has been defined, enter a lower (more critical) value to ensure that the generator will only start automatically when absolutely necessary.

- Stop when condition is met for: This option enters a time interval before stopping the generator when the "stop" value is reached. This ensures that the voltage rise is stable.

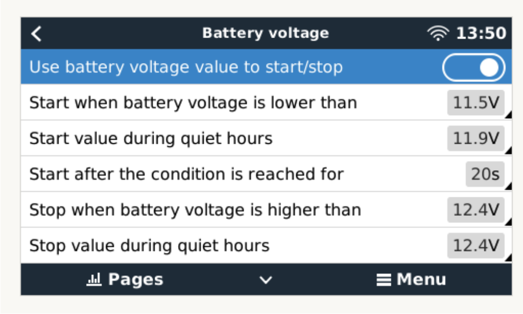

Figure S11. Battery voltage configuration window.

AC load triggered relays (Figure S12):

The AC load triggers function similarly to other triggers, but the function is refined by a Metering setting. This Metering setting is available in firmware version v2.0 and later and has three possible values:

Total Consumption (Default option)

Total AC output of the inverter

Highest phase of inverter AC output

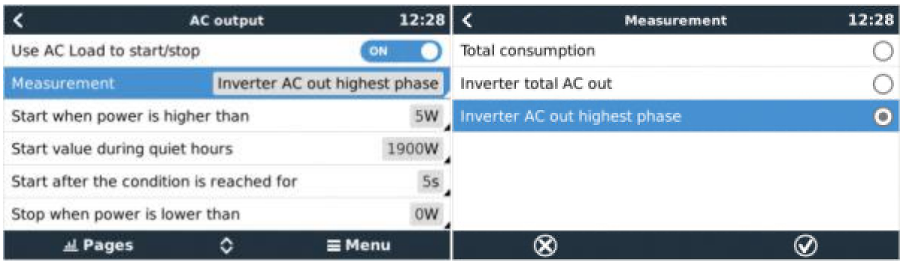

Figure S12. Battery AC power configuration window.

Inverter high temperature triggered relays (Figure S13):

- Start if there is a high temperature warning: This option can be set to on or off.

- Start when there is an active warning for: Set a time interval to avoid temporary temperature rises, caused by high but brief AC demands.

- Once the warning has disappeared, stop after: Set a time interval to ensure that the temperature reduction of the inverter - usually caused by lower power demand - is stable.

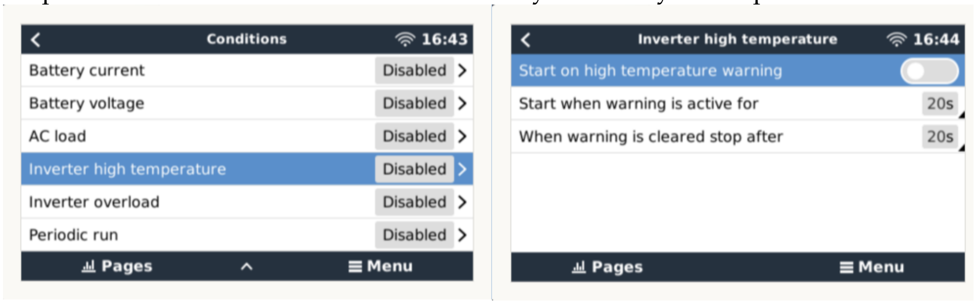

Figure S13. Inverter high temperature configuration window sequence.

Inverter overload triggered relays (Figure S14):

- Start when there is an overload warning: This option can be set to On or Off.

- Start when there is an active warning for: Set a time interval to ensure that the inverter overload warning is not due to a short duration high AC demand.

- Once the warning has disappeared, stop after: Set a time interval to ensure that the AC demand reduction is stable.

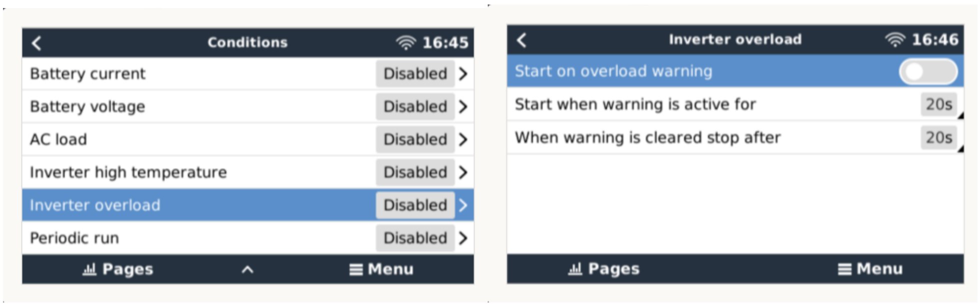

Figure S14. Inverter overload configuration window sequence.

From the initial configuration selected in Figure S15, start/stop of generation, the relay function could also be used in conjunction with a connected external sensor to perform disconnections in the event of a critical temperature increase due to the occurrence of a runaway effect in the battery or in an inverter.

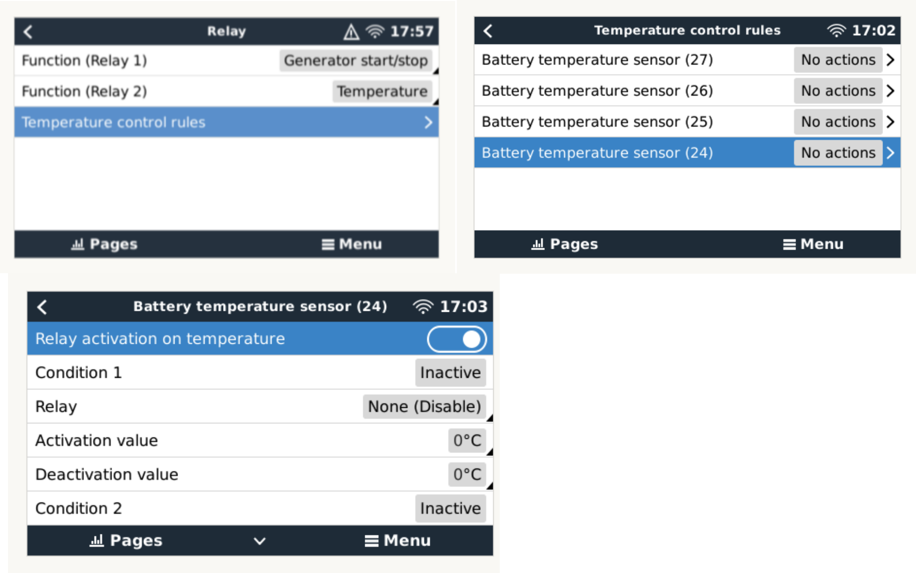

Figure S15. Sequence of activation of temperature control rules.

Another option is to use the additional integrated relay provided in the monitor of the BMV-712 smart device to be used for system fail-safe actions. This relay can be configured to act on all of the relay parameters listed in the left column of Table 1.

Figure 1 shows the appropriate options of the relay set installation:

- Starting and stopping of the generator system driven by Relay 1 integrated in the Cerbo GX, due to the threshold values reached by the V, I and SOC parameters of the battery.

- Start and stop of the generator system driven by Relay 2 integrated in the Cerbo GX, due to the threshold values reached by the Temperature parameter obtained from the temperature sensor 24 connected to the negative pole of the battery.

- Starting and stopping of the generator system driven by Relay 2 integrated in the Cerbo GX, due to the threshold values reached by the Temperature parameter obtained from the sensor 25 connected to the negative pole of the inverter.

- Connection and disconnection and isolation of the battery by actuating the relay integrated in the BMS monitor, which in turn acts on the two power relays.

Supplementary S3: BMS parameters

Table S1. Battery Temperature & Electrical Parameters.

Electrical Specification	Value
Design floating Life @ 20°C (68°F)	12 years
Nominal Capacity @ 25°C /77°F	20 hour rate 7.88 A to 1.80Vpc: 158 Ah<br>10 hour rate 15.0 A to 1.80Vpc: 150 Ah<br>5 hour rate 26.3 A to 1.75Vpc: 132Ah<br>1 hour rate 97.5A to 1.60Vpc: 97.5Ah
Internal Resistance	(Fully charged battery @ 25°C /77°F) 3.9mΩ
Max. Discharge Current @ 25°C /77°F	1000A (5S)
Charge Methods:	Constant voltage charge @ 25°C/77°F<br>Cycle Use 14.4 ~15.0V<br>Max. Current 37.5A<br>Standby Use 13.5V~13.8V
Operating Temperature Range	Discharge: -20 ~ 55°C<br>Charge: 0 ~ 40°C<br>Storage: -20 ~ 55°C<br>Recommended Operating Temp.: 20~25°C
Self-Discharge	The capacity declines by 3% per month@ 20°C (68°F). The batteries can be stored up to 6 months @ 25°C (77°F) and then a freshening charge is required. The interval under higher temperature would be shorter.

Table S2. Inverter XJC20001255U Temperature & Electrical Parameters.

Parameter	Value
Rated / Peak AC Output Power	2000/4000W
AC Output Current	8.70ACA
AC Output Voltage / Frequency	230VAC / 50Hz
AC Output Waveform	Pure Sine Wave (THD<3%)
Peak Efficiency	>92%.
Nominal DC Input Voltage	12.50VDC
Operating DC Input Voltage Range	10.50 - 15.50VDC
Low Voltage Shutdown	10.50VDC (Programmable to 11.80VDC for car starter battery)
Low Voltage Reclosing	12.00VDC (Programmable to 12.60VDC for car starting battery)
Over Voltage Shutdown	15.50VDC
Charge Current (max)	55ACC
Battery Type	AGM, GEL, Flooded
Minimum Battery Capacity	> 480Ah - 12V
Operating Temperature	0°C ~ +40°C ~ +40°C (+32°F ~ +104°F)
Storage Temperature	-20°C ~ +60°C (-4°F ~ +140°F)

Table S3. Relay data.

Module description:<br>1. Maximum load on normally open pin: AC 250V/10A, DC 30V/10A.<br>2. Trigger current 5mA;<br>3. Module can be set high level or low level trigger by jumper wire;<br>4. Power indicator light (green), relay status indicator light (red);<br>5. Module size: 50mm*41mm*18.5mm.
Module interface specifications:<br>1. DC+ : DC power supply positive pole<br>2. DC- : DC power supply negative pole<br>3. IN1 : channel 1 signal triggering pin<br>4. IN2 : channel 2 signal triggering pin
Relay output:<br>1. Normally closed pin (NC1) : channel 1 relay normally closed pin<br>2. Common pin (COM1) : channel 1 relay common pin<br>3. Normally opened pin (NO1) : channel 1 relay normally opened pin<br>4. Normally closed pin (NC2) : channel 2 relay normally closed pin<br>5. Common pin (COM2) : channel 2 relay common pin<br>6. Normally opened pin (NO2) : channel 2 relay normally opened pin
High or low level trigger option:<br>1. S1: It is option switch for channel 1 relay;<br>2. S2: It is option switch for channel 2 relay;<br>3. It is low level triger if COM pin connect to LOW pin; It is high level triger if COM connect to High.

Table S4. BMV-712 smart parameters

01. Battery capacity
Battery capacity in Ampere hours
Default Range Progression step 200 Ah 1 - 9,999 Ah 1 Ah

02. Charged Voltage
The battery voltage must be above this voltage level to consider the battery as fully charged. The Charged Voltage parameter should always be slightly below the end-of-charge voltage (typically 0.2 or 0.3 V below the charger "float" voltage), see section 3.7 for recommended settings.
Range Progression step
0-70V 0.1

03. Tail current
Once the charging current has fallen below the set tail current (expressed as a percentage of the battery capacity), the battery is considered fully charged.
Note: Some battery chargers stop charging when the current drops below a predetermined threshold. The tail current must be above this threshold.
Default Range Progression step
4 % 0,5 - 10 % 0,1 %

04. Charged detection time
This is the time during which the charging parameters (Charged voltage and Tail current) must be reached to consider the battery fully charged.
Default Range Progression step
3 min 1 - 50 min 1 min

---

05. Peukert exponent
If unknown, it is recommended to keep this value at 1.25 (default) for lead-acid batteries and change it to 1.05 for Li-Ion batteries. A value of 1.00 disables Peukert compensation.
Default Range Progression step
1.25 1 - 1.5 0.01

---

06. Charge Efficiency Factor
The Charge Efficiency Factor compensates for Ah losses that may occur during charging.
100 % means no loss has occurred.
Default Range Progression step
95 % 50 - 100 % 1 %

---

07. Current threshold
When the measured current falls below this value, it will be considered zero.
The current threshold is used to cancel very low currents that may negatively affect the long-term readings of the state of charge in noisy environments. For example, if the actual long-term current is 0.0 A., and due to small noises or mismatches the battery monitor measures - 0.05 A., the BMV may eventually erroneously indicate that the battery needs to be charged. When the current threshold in this example is set to 0.1, the BMV calculates based on 0.0 A. to eliminate errors. A value of 0.0 disables this function.
Default Range Progression step
0.1 TO 0 - 2 TO 0.01 A

---

08. Time-to-go averaging period
Specifies the time window (in minutes) that the moving average filter works with. A value of 0 disables the filter and provides an instantaneous (real-time) reading; however, the displayed values may fluctuate greatly. Selecting the longer time period (12 minutes) ensures that only long-term load fluctuations are included in the remaining autonomy calculations.
Default Range Progression step
3 min. 0 - 12 min. 1 min.

---

09. Zero current calibration
If the BMV reads a non-zero current even without a load connected, and the battery is not being charged, this option can be used to calibrate the zero reading.
Make sure that there is actually no current flowing in the battery (disconnect the cable between the load and the shunt), then press SELECT.

---

10. Synchronise
This option can be used to manually synchronize the BMV.
Press SELECT to synchronize.
The BMV can also be synchronized while in normal operating mode by holding down the + and - buttons simultaneously for 3 seconds.

Table S5. Battery settings.

01. Battery capacity<br>Battery capacity in amp hours<br>Default Step size<br>200 Ah Range<br>Range Step size<br>1 – 9999 Ah<br>Step size<br>1 Ah
02. Charged voltage<br>The battery voltage must be above this voltage level to consider the battery as fully charged.<br>The charged-voltage-parameter should always be slightly below the end of charge voltage of the charger (usually 0.2 V or 0.3 V below the 'float' voltage of the charger).<br>See section 3.7 for recommended settings.<br>BMV-712 Smart<br>Default Range Step size<br>See table, sect 3.7 0 – 70 V 0.1 V<br>Range<br>0 – 70 V<br>Step size<br>0.1 V
03. Tail current<br>Once the charge current has dropped to less than the set tail current (expressed as percentage of the battery capacity), the battery is considered as fully charged.<br>Remark:<br>Some battery chargers stop charging when the current drops below a set threshold. The tail current must be set higher than this threshold.<br>Default Range Step size<br>4 % 0.5 – 10 % 0.1 %
04. Charged detection time<br>This is the time the charged-parameters (Charged voltage and Tail current) must be met in order to consider the battery fully charged.<br>Default. Range Step size<br>3 minutes 1 – 50 minutes 1 minute
05. Peukert exponent<br>When unknown it is recommended to keep this value at 1.25 (default) for lead acid batteries and change to 1.05 for Li-ion batteries. A value of 1.00 disables the Peukert compensation.<br>Default Range Step size<br>1.25 1-1.5 0.01
06. Charge Efficiency Factor<br>The Charge Efficiency Factor compensates for the Ah losses during charging.<br>100 % means no loss.<br>Default Range Step size<br>95% 50-100% 1%
07. Current threshold<br>When the current measured falls below this value it will be considered zero.<br>The current threshold is used to cancel out very small currents that can negatively affect the long term state of charge readout in noisy environments. For example if the actual long term current is 0.0 A and due to injected noise or small offsets the battery monitor measures 0.05 A, and in the long term the BMV can incorrectly indicate that the battery needs recharging. When the current threshold in this example is set to 0.1 A, the BMV calculates with 0.0 A so that errors are eliminated.<br>A value of 0.0 A disables this function.<br>Default Range Step size<br>0.5 A 0-2 A 0.01 A
08. Time-to-go averaging period<br>Specifies the time window (in minutes) that the moving averaging filter works.<br>A value of 0 disables the filter and gives an instantaneous (real-time) readout; however the displayed value may fluctuate heavily. Selecting the longest time (12 minutes) ensures that only long term load fluctuations are included in the time-to-go calculations.

Default Range Step size<br>3 minutes 0-12 minutes 1 minute
09. Zero current calibration<br>If the BMV reads a non-zero current even when there is no load and the battery is not being charged, this option can be used to calibrate the zero reading.<br>Ensure that there really is no current flowing into or out of the battery (disconnect the cable between the load and the shunt), then press SELECT.
10. Synchronise<br>This option can be used to manually synchronise the BMV.<br>Press SELECT to synchronise.<br>The BMV can also be synchronised when in normal operating mode by holding the + and – buttons simultaneously for 3 seconds.

Table S6. Relay settings.
Remark: thresholds are disabled when set at 0

11. Relay mode<br>DFLT Default mode. The relay thresholds Nos. 16 up to 31 can be used to control the relay.<br>CHRG Charger mode. The relay will close when the state of charge falls below setting 16 (discharge floor) or when the battery voltage falls below setting 18 (low voltage relay).<br>The relay will be open when the state of charge is higher than setting 17 (clear state of charge relay) and the battery voltage is higher than setting 19 (clear low voltage relay).<br>Application example: start and stop control of a generator, together with settings 14 and 15.<br>REM Remote mode. The relay can be controlled via the VE.Direct interface. Relay settings 12 and 14 up to 31 are ignored as the relay is under the full control of the device connected via the VE.Direct interface.
12. Invert relay<br>This function enables selection between a normally de-energised (contact open) or a normally energised (contact closed) relay. When inverted, the open and closed conditions as described in setting 11 (DFLT and CHRG), and settings 14 up to 31 are inverted.<br>The normally energized setting will slightly increase supply current in the normal operating mode.<br>Default Range<br>OFF: Normally de-energised OFF: Normally de-energised / ON: normally energised
13. Relay state (read only)<br>Displays whether the relay is open or closed (de-energised or energised).<br>Range<br>OPEN/CLSD
14. Relay minimum closed time<br>Sets the minimum amount of time that the CLOSED condition will remain present after the relay has been energised. (changes to OPEN and de-energised if the relay function has been inverted)<br>Application example: set a minimum generator run time (relay in CHRG mode).
15. Relay-off delay<br>Sets the amount of time the 'de-energise relay' condition must be present before the relay opens.<br>Application example: keep a generator running for a while to better charge the battery (relay in CHRG mode).<br>Default Range Step size<br>0 minutes 0-500 minutes 1 minute
16. SoC relay (Discharge floor)<br>When the state of charge percentage has fallen below this value, the relay will close.<br>The time-to-go displayed is the time to reach the discharge floor.<br>Default Range Step size<br>50 % 0-99 % 1%
17. Clear SoC relay<br><br>When the state of charge percentage has risen above this value, the relay will open (after a delay, depending on setting 14 and/or 15). This value needs to be greater than the previous parameter setting. When the value is equal to the previous parameter the state of charge percentage will not close the relay.

Default Range Step size<br>90 % 0-99 % 1%
18. Low voltage relay<br>When the battery voltage falls below this value for more than 10 seconds the relay will close.
19. Clear low voltage relay<br>When the battery voltage rises above this value, the relay will open (after a delay, depending on setting 14 and/or 15). This value needs to be greater than or equal to the previous parameter.
20. High voltage relay<br>When the battery voltage rises above this value for more than 10 seconds the relay will close.
21. Clear high voltage relay<br>When the battery voltage falls below this value, the relay will open (after a delay, depending on setting 14 and/or 15). This value needs to be less than or equal to the previous parameter.<br>BMV-700 / BMV-702 / BMV 712 Smart<br>Default Range Step size<br>0 V 0 – 95 V 0.1 V
22. Low starter voltage relay -702 and -712 only<br>When the auxiliary (e.g. starter battery) voltage falls below this value for more than 10 seconds the relay will be activated.
23. Clear low starter voltage relay -702 and -712 only<br>When the auxiliary voltage rises above this value, the relay will open (after a delay, depending on setting 14 and/or 15). This value needs to be greater than or equal to the previous parameter.
24. High starter voltage relay -702 and -712 only<br>When the auxiliary (e.g. starter battery) voltage rises above this value for more than 10 seconds, the relay will be activated.
25. Clear high starter voltage relay -702 and -712 only<br>When the auxiliary voltage falls below this value, the relay will open (after a delay, depending on setting 14 and/or 15). This value needs to be less than or equal to the previous parameter.<br>Default Range Step size<br>0 V 0 – 95 V 0.1 V
26. High temperature relay -702 and -712 only<br>When the battery temperature rises above this value for more than 10 seconds, the relay will be activated.
27. Clear high temperature relay -702 and -712 only<br>When the temperature falls below this value, the relay will open (after a delay, depending on setting 14 and/or 15). This value needs to be less than or equal to the previous parameter.
28. Low temperature relay -702 and -712 only<br>When the temperature falls below this value for more than 10 seconds, the relay will be activated.
29. Clear low temperature relay -702 and -712 only<br>When the temperature rises above this value, the relay will open (after a delay, depending on setting 14 and/or 15). This value needs to be greater than or equal to the previous parameter.<br>See setting 67 for choosing between °C and °F.<br>Default Range Step size<br>0 °C -40 – 99 °C 1 °C<br>0 °F -40 – 210 °F 1 °F
30. Mid voltage relay -702 and -712 only<br>When the midpoint voltage deviation rises above this value for more than 10 seconds, the relay will be activated. See section 5.2 for more information about the midpoint voltage.
31. Clear mid voltage relay -702 and -712 only<br>When the midpoint voltage deviation falls below this value, the relay will open (after a delay, depending on setting 14 and/or 15). This value needs to be less than or equal to the previous parameter.<br><br>Default Range Step size<br>0 % 0 – 99 % 0.1 %

Table S7. Alarm-Buzzer settings.
Remark: thresholds are disabled when set at 0

32. Alarm buzzer
When set, the buzzer will sound an alarm. After a button is pressed the buzzer will stop sounding. When disabled the buzzer will not sound an alarm.

Default	Range
ON	ON/OFF

33. Low SoC alarm
When the state of charge falls below this value for more than 10 seconds the low SoC alarm is turned on. This is a visual and audible alarm. It does not energise the relay.

34. Clear low SoC alarm
When the state of charge rises above this value, the alarm is turned off. This value needs to be greater than or equal to the previous parameter.

Default	Range	Step size
0 %	0 – 99 %	0.1 %

35. Low voltage alarm
When the battery voltage falls below this value for more than 10 seconds the low voltaje alarm is turned on. This is a visual and audible alarm. It does not energise the relay.

36. Clear low voltage alarm
When the battery voltage rises above this value, the alarm is turned off. This value needs to be greater than or equal to the previous parameter.

37. High voltage alarm - When the battery voltage rises above this value for more than 10 seconds the high voltage alarm is turned on. This is a visual and audible alarm. It does not energise the relay.

38. Clear high voltage alarm - When the battery voltage falls below this value, the alarm is turned off. This value needs to be less than or equal to the previous parameter.
BMV-700 / BMV-702 / BMV 712 Smart

Default	Range	Step size
0 V	0 – 95 V	0.1 V

39. Low starter voltage alarm -702 and -712 only
When the auxiliary (e.g. starter battery) voltage falls below this value for more than 10 seconds the alarm will be activated. This is a visual and audible alarm. It does not energise the relay.

40. Clear low starter voltage alarm -702 and -712 only
When the auxiliary voltage rises above this value, the alarm is switched off. This value needs to be greater than or equal to the previous parameter.

41. High starter voltage alarm -702 and -712 only
When the auxiliary (e.g. starter battery) voltage rises above this value for more than 10 seconds, the alarm will be activated. This is a visual and audible alarm. It does not energise the relay.

42. Clear high starter voltage alarm -702 and -712 only
When the auxiliary voltage falls below this value, the alarm is switched off. This value needs to be less than or equal to the previous parameter.

Default	Range	Step size
0 V	0 – 95 V	0.1 V

43. High temperature alarm -702 and -712 only
When the battery temperature rises above this value for more than 10 seconds, the alarm will be activated. This is a visual and audible alarm. It does not energise the relay.

44. Clear high temperature alarm -702 and -712 only
When the temperature falls below this value, the alarm is switched off. This value needs to be less than or equal to the previous parameter.

45. Low temperature alarm -702 and -712 only
When the temperature falls below this value for more than 10 seconds, the alarm will be activated. This is a visual and audible alarm. It does not energise the relay.

46. Clear low temperature alarm -702 and -712 only
When the temperature rises above this value, the alarm is switched off. This value needs to be greater than or equal to the previous parameter.
See parameter 67 for choosing between °C and °F.

Default	Range	Step size
0 °C	-40 – 99 °C	1 °C
0 °F	-40 – 210 °F	1 °F

47. Mid voltage alarm -702 and -712 only<br>When the midpoint voltage deviation rises above this value for more than 10 seconds, the alarm will be activated. This is a visual and audible alarm. It does not energise the relay.<br>See section 5.2 for more information about midpoint voltage.<br>Default Range Step size<br>2 % 0 – 99 % 0.1 %
48. Clear mid voltage alarm -702 and -712 only<br>When the midpoint voltage deviation falls below this value, the alarm is switched off. This value needs to be less than or equal to the previous parameter.<br>Default Range Step size<br>1.5 % 0 – 99 % 0.1 %

Table S8. Display settings.

49. Backlight intensity<br>The intensity of the backlight, ranging from 0 (always off) to 9 (maximum intensity)<br>Default Range Step size<br>5 0-9 1
50. Backlight always on<br>When set the backlight will not automatically turn off after 60 seconds of inactivity.<br>Default Range<br>OFF OFF/ON
51. Scroll speed<br>The scroll speed of the display, ranging from 1 (very slow) to 5 (very fast).<br>Default Range Step size<br>2 1 – 5 1
52. Main voltage display<br>Must be ON to display the voltage of the main battery in the monitoring menu.
53. Current display<br>Must be ON to display current in the monitoring menu.
54. Power display<br>Must be ON to display power in the monitoring menu.
55. Consumed Ah display<br>Must be ON to display consumed Ah in the monitoring menu.
56. State of charge display<br>Must be ON to display state of charge in the monitoring menu.
57. Time-to-go display<br>Must be ON to display time-to-go in the monitoring menu.
58 Starter voltage display -702 and -712 only<br>Must be ON to display the auxiliary voltage in the monitoring menu.
59. Temperature display -702 and -712 only<br>Must be ON to display the temperature in the monitoring menu.
60. Mid-voltage display -702 and -712 only<br>Must be ON to display the midpoint voltage in the monitoring menu.<br>Default Range<br>ON ON/OFF

Table S9. Miscellaneous.

61. Software version (read only)<br>The software version of the BMV
62. Restore defaults<br>Resets all settings to factory default by pressing SELECT.<br>When in normal operating mode, factory settings can be restored by pressing SETUP and SELECT simultaneously for 3 seconds (only if setting 64, Lock setup, is off).

63. Clear history
Clears all history data by pressing SELECT.

58 Starter voltage display -702 and -712 only
Must be ON to display the auxiliary voltage in the monitoring menu.

59. Temperature display -702 and -712 only
Must be ON to display the temperature in the monitoring menu.

60. Mid-voltage display -702 and -712 only
Must be ON to display the midpoint voltage in the monitoring menu.

Default	Range
ON	ON/OFF

64. Lock setup
When on, all settings (except this one) are locked and cannot be altered.

Default	Range
OFF	OFF/ON

65. Shunt current
When using a shunt other than the one supplied with the BMV, set to the rated current of the shunt.

Default	Range	Step size
500 A	1 – 9999 A	1 A

66. Shunt voltage
When using a shunt other than the one supplied with the BMV, set to the rated voltage of the shunt.

Default	Range	Step size
50 mV	1 mV– 75 mV	1 mV

67. Temperature unit
CELC Displays the temperature in °C.
FAHR Displays the temperature in °F.

Default	Range
CELC	CELC/FAHR

68. Temperature coefficient
This is the percentage the battery capacity changes with temperature, when temperature decreases to less than 20 °C (above 20 °C the influence of temperature on capacity is relatively low and is not taken into account). The unit of this value is “ %cap/°C” or percent capacity per degree Celsius. The typical value (below 20 °C) is 1 %cap/°C for lead acid batteries, and 0.5 %cap/°C for Lithium Iron Phosphate batteries.

Default	Range	Step size
0 %cap/°C	0 – 2 %cap/°C	0.1 %cap/°C

69. Aux input
Sets the function of the auxiliary input:
NONE Disables the auxiliary input (default)
START Auxiliary voltage, e.g. a starter battery.
MID Midpoint voltage.
TEMP Battery temperature.
The cable with integrated temperature sensor has to be purchased separately (part no: ASS000100000). This temperature sensor is not interchangeable with other Victron temperature sensors, as provided with Multis or battery chargers.

70. Start synchronised
When ON, the BMV will consider itself synchronised when powered-up, resulting in a state of charge of 100 %. If set to OFF, the BMV will consider it unsynchronised when powered-up, resulting in a state of charge that is unknown until the first actual synchronisation.

Default	Range
ON	OFF/ON

71. Bluetooth mode (BMV-712 only)
Determines whether to enable Bluetooth. If turned OFF using the VictronConnect app, the Bluetooth functionality is not disabled until disconnected from the BMV. Note that this setting is only available when the firmware of the on-board Bluetooth module supports this functionality.

Default	Range
ON	OFF/ON
